# Sex-dimorphic DNA lesion accumulation drives a PDK4 metabolic checkpoint in skeletal muscle

**DOI:** 10.21203/rs.3.rs-10972321/v1

**Published:** 2026-10-06

**Authors:** Sriravali Pulipaka, Akanksha Sachan, Zachary Clemens, Kritika Chadda, Jagruti Kosaraju, Ulalume Hernández-Arciga, Zarifeh Heidari Rarani, Augustine George, Emily Madigan, Lillian Williams, Naghmeh Abbasi, Isabel Joyce, Bommana Raghunath Reddy, Kritika Rao, Savita Khanna, Chandan K Sen, Sruti Shiva, Andrey A Parkhitko, Eric S Goetzman, Michael J Jurczak, Daniel J Shiwarski, Natalie R Gassman, Jishnu Das, Amrita Sahu, Aditi U Gurkar

**Affiliations:** 1 Aging Institute, University of Pittsburgh School of Medicine, University of Pittsburgh.; 2 Division of Geriatric Medicine, University of Pittsburgh School of Medicine; 3 Centre for Systems Immunology and Departments of Immunology and Computational and Systems Biology.; 4 The Joint CMU-Pitt Ph.D. Program in Computational Biology, Carnegie Mellon University and University of Pittsburgh.; 5 McGowan Institute for Regenerative Medicine, Department of Surgery, University of Pittsburgh.; 6 Center for Metabolism and Mitochondrial Medicine, Department of Medicine, University of Pittsburgh.; 7 Division of Endocrinology and Metabolism, Department of Medicine, University of Pittsburgh.; 8 Vascular Medicine Institute, University of Pittsburgh.; 9 Department of Bioengineering, Swanson School of Engineering, University of Pittsburgh.; 10 Department of Paediatrics, University of Pittsburgh.; 11 Department of Pharmacology and Toxicology, University of Alabama at Birmingham.; 12 Department of Physical Medicine and Rehabilitation, University of Pittsburgh.

## Abstract

Skeletal muscle loss and functional decline are sexually dimorphic, with fast-twitch type II fibers disproportionately vulnerable to age-related atrophy. DNA damage accumulation is a hallmark of aging whose consequences in myofibers remain poorly understood. Using a type II fiber-specific DNA repair-deficient mouse, we show that the same genotoxic insult produces qualitatively different lesion landscapes by sex, oxidative damage in females and uracil misincorporation in males. Females exhibited greater atrophy pathway activation, paralleling clinical evidence that genotoxic stress disproportionately drives frailty in women. Single-nucleus RNA sequencing and metabolomics identified PDK4 as the key female-specific metabolic driver. Pharmacologic PDK4 inhibition restored oxidative metabolism and myotube health. Dietary α-ketoglutarate, replenishing TCA intermediates downstream of the PDK4 block, rescued contractile force and metabolic profiles in females. Human aging data confirmed conservation of this pathway, revealing a sex-specific mechanism linking DNA damage to metabolic dysfunction and identifying an accessible therapeutic strategy to combat female-predominant frailty.

## Introduction

Skeletal muscle (SKM) accounts for 40–50% of body mass making it the body's primary metabolic organ. Its deterioration independently predicts hospitalization, disability, institutionalization, and all-cause mortality[1-7]. This decline begins in middle age; muscle mass falls 1%/year from age 30–50, accelerating to >3%/year beyond the seventh decade[8, 9]. The midlife trajectory already forecasts late-life outcomes. Those with the lowest relative muscle power carry a nearly six-fold greater mortality hazard than those with the highest,[10] and midlife grip strength prospectively predicts old-age disability[11]. In older adults, gait speed outperforms age, sex, chronic conditions, BMI, and hospitalization combined as a survival predictor [12] and muscle strength is inversely associated with cardiovascular, respiratory, cancer, and infectious disease mortality across 28 countries[2, 13-16]Despite this epidemiology spanning midlife to advanced age, the intracellular mechanisms initiating loss of muscle resilience, and why trajectories diverge so dramatically between individuals, remain poorly understood, blocking early, targeted intervention.

SkM does not deteriorate uniformly across sexes, and this dimorphism carries profound consequences for aging, frailty, and therapeutic vulnerability[17-19]. Men experience greater absolute loss of muscle mass, while women sustain disproportionate impairment of functional performance, with markedly higher rates of frailty, fall-related injury, and loss of independence at comparable age[20-26]. Female cancer survivors treated with genotoxic chemotherapy exemplify this vulnerability at accelerated timescale: they exhibit premature frailty at rates nearly three times those of male survivors (44.6% vs. 15.6%), and experience physical function decline persisting beyond two years post-treatment, a trajectory characterized as accelerated biological aging[27-32]. Despite this epidemiological clarity, the intracellular mechanisms that make female muscle disproportionately susceptible to functional deterioration remain largely uncharacterized, representing a critical and underexplored gap in the biology of aging. Central to this sex dimorphism is the selective vulnerability of fast-twitch type II myofibers. Males possess greater type II fiber cross-sectional areas and a higher proportion of these fibers relative to females, a structural advantage that matters because type II fibers generate maximal contractile force, sustain power output, and are disproportionately depleted with age. The mechanisms initiating type II fiber decline, and why this decline is accelerated in females, are unknown.

DNA damage accumulation is a hallmark of aging whose contribution to muscle decline has been largely overlooked and never examined in fast-twitch type II fibers. Every major study of DNA damage in aging muscle has been conducted in muscle stem cells (MuSC), leaving the most vulnerable fiber type wholly uncharacterized. This blind spot is compounded by a key vulnerability of postmitotic cells: dramatically reduced DNA repair capacity relative to proliferating cells causes lesions to persist[33-37]. In 146 healthy humans, oxidative DNA lesion burden increased 25% with age and correlated directly with mitochondrial ATP production and gait speed, underscoring functional relevance[38]. Critically, not all lesions are equivalent; oxidative 8-oxoguanine (8oxoG) and uracil misincorporation engage distinct repair pathways, as well as may impose different transcriptional and metabolic demands. Whether male and female muscle accumulates different lesion types, and what the downstream consequences of that difference might be, has never been examined. The field has implicitly treated DNA damage as a monolithic stressor scaling linearly with burden; whether lesion quality shapes sex-specific aging trajectories in postmitotic muscle remains entirely unknown. Here, we address these gaps through an integrated, sex-stratified investigation of DNA lesion type, downstream metabolic reprogramming, and functional consequence in type II skeletal muscle fibers.

## RESULTS

### Type II fiber-specific *Ercc1* loss reveals sex-dimorphic DNA lesion accumulation in skeletal muscle

To model age-relevant stress in fast-twitch (type II) skeletal muscle fibers, the fiber type preferentially lost with aging, we generated a cell type specific *Ercc1* knockout model (SkM-KO) by combining CRISPR-Cas9 conditional *Ercc1* mice with *Myl1-Cre* drivers (Fig. 1A, Supp Fig 1A). Efficient *Ercc1* deletion in type II fiber was confirmed by both, qPCR and BaseScope analysis, each demonstrating reduced expression in SkM-KO muscle compared to controls (Fig. 1B-C, Supp Fig. 1B). Deletion was confirmed to be restricted to type II fiber-containing muscle [tibialis anterior (TA)], as *Ercc1* expression was preserved in the soleus, a type I-dominant muscle, and in liver (Fig. 1B).

To comprehensively map the spectrum of DNA damage accumulating in SkM-KO type II muscle, we employed the Repair Assisted Damage Detection (RADD) assay, a sensitive, lesion-specific platform that leverages bacterial repair enzymes to convert diverse DNA lesions into quantifiable signals at single-nucleotide resolution. Using three enzymes targeting distinct damage classes: oxRADD for oxidative lesions, T4 PDG for UV-induced pyrimidine dimers, and UDG for uracil-containing lesions, we uncovered a striking and unexpected sexual dimorphism in the landscape of accumulated DNA damage. Female SkM-KO mice exhibited significant elevations in oxidative and pyrimidine dimer-associated lesions relative to controls, while uracil lesions remained unchanged. Males showed a diametrically opposite profile: uracil-associated damage was significantly increased, whereas oxidative and pyrimidine dimer lesions were unaffected (Fig. 1D). The co-elevation of T4PDG signal alongside OxRADD specifically in females indicates that the full spectrum of ERCC1-dependent lesion classes accumulates in female myofibers upon repair deficiency. This broad NER-dependent lesion accumulation, encompassing both oxidative adducts and bulky pyrimidine lesions, suggests that female type II myofibers generate a wider range of endogenous genotoxic substrates than males under the same conditions, or that the threshold for lesion generation is lower in the female myonuclear environment.

To determine whether the sex-dimorphic lesion landscape observed in SkM-KO mice reflects physiological aging biology, we performed longitudinal RADD profiling in naturally aged male and female mice across three timepoints spanning 4 to 28 months of age. Female type II myofibers showed progressive and significant accumulation of oxidative and bulky lesions, with signals rising monotonically through 28 months, precisely mirroring the SkM-KO female lesion profile at accelerated timescale (Supp Fig. 1C). Male myofibers showed a qualitatively different trajectory: oxidative lesion accumulation was delayed and modest, emerging significantly only at 28 months, while UDG signal showed early elevation followed by apparent plateau, consistent with active containment of uracil lesions through aging (Supp Fig. 1C). Notably, male control myofibers exhibited higher basal OxRADD signal than females but showed no further increase following ERCC1 loss, suggesting a mechanism that limits oxidative lesion accumulation despite impaired repair. This may reflect enhanced oxidative damage buffering through base excision repair, antioxidant capacity, or androgen-mediated ROS suppression. In contrast, females, despite lower basal oxidative damage, lack this buffering capacity and progressively accumulate oxidative lesions when ERCC1-mediated repair is compromised. These data establish that the sex-dimorphic lesion landscape is a progressive, lifelong feature of type II myofiber biology in physiological aging, not a repair deficiency artifact, and that the SkM-KO model faithfully recapitulates the female aging lesion trajectory at accelerated pace. Together, these data identify biological sex as an important determinant of the source and nature of DNA damage accumulating in postmitotic type II myofibers, providing new insight into the mechanisms underlying muscle aging.

This sex-dependent divergence in DNA damage landscapes was mirrored, and further elaborated, at the level of muscle structure. Female SkM-KO mice showed no significant differences in CSA, or fiber-type distribution relative to controls (Fig. 1E, Supp Fig. 2A) a structural profile that appeared, paradoxically, largely intact. By contrast, male SkM-KO muscle showed significant reduction in CSA, indicative of frank atrophy, and fiber-type analysis revealed a selective loss of Type IIb fibers, consistent with preferential vulnerability of the fastest, most glycolytic myofiber population to genotoxic stress (Fig. 1E). This atrophic remodelling of SkM-KO, occurred without fibrotic collagen deposition in either sex (Fig 1E, Supp Fig. 2A). In addition, no such structural differences were observed in of mixed fiber type muscle compartment, quadriceps and purely type I muscle Soleus (Supp Fig 2B-C). The absence of structural alterations in the quadriceps and soleus provides two independent specificity controls. Soleus preservation confirms that *Myl1-Cre*-mediated ERCC1 deletion is restricted to type II fibers, excluding non-cell-autonomous effects on type I fibers. In contrast, preserved quadriceps despite their type II fiber content suggests that metabolic demand, rather than fiber type alone, determines when ERCC1 loss becomes pathogenic. These findings support a model in which genotoxic stress drives dysfunction specifically under high metabolic load, providing a mechanistic basis for the selective impairment of activities requiring sustained type II fiber recruitment.

### Type II fiber genotoxic stress produces opposite structural and functional trajectories by sex, revealing a structural-functional uncoupling that implicates sex-specific cell-intrinsic mechanisms.

Given the marked sex-specific differences in muscle structure, we next assessed body composition and physical function. Female SkM-KO mice showed no significant changes in body weight, body composition or food intake relative to controls (Fig 2A-B). By contrast, male SkM-KO mice exhibited increased fat mass and reduced lean mass despite unchanged body weight (Fig 2A), indicating lean-to-fat tissue replacement consistent with myosteatosis. This phenotype aligns with the selective type IIB fiber atrophy observed in Fig. 1 and mirrors myosteatosis reported in human sarcopenia and chemotherapy-induced muscle wasting. Food intake remained unchanged in both female and male SkM-KO mice relative to their respective controls (Fig. 2B).

Type II fiber-specific ERCC1 loss produced strikingly divergent trajectories of functional and structural decline between sexes- a dissociation that challenges the assumption that muscle mass predicts functional capacity. Despite showing minimal structural deterioration, female SkM-KO mice sustained the most pronounced functional impairment. Females exhibited significant reductions in total activity, distance travelled, and grip strength relative to controls, accompanied by decreased latency to fall on rotarod (Fig. 2C-D). These deficits span the spectrum of type II fiber-dependent function: grip strength and rotarod performance require rapid force generation, while locomotor activity reflects sustained type II fiber engagement. Their coordinated impairment in structurally intact muscle indicates that genotoxic stress in type II fibers disrupts their functional capacity through a mechanism independent of fiber atrophy, pointing to intrinsic metabolic or contractile machinery failure rather than simple fiber loss. Despite greater structural pathology, including type IIB fiber loss and reduced CSA, male SkM-KO mice largely preserved grip strength and rotarod performance, with only modest reductions in locomotor activity (Fig 2C-D). This dissociation suggests that the remaining fibers retain sufficient contractile capacity and metabolic flexibility to sustain function, consistent with the distinct lesion profile in males (uracil misincorporation), which is less likely to disrupt transcription or drive metabolic reprogramming. Thus, the male phenotype reflects structural atrophy without substantial functional failure.

This functional dissociation was further substantiated by direct assessment of intrinsic contractile properties. Female SkM-KO mice exhibited reduced tetanic force and a rightward shift in the force-frequency relationship, requiring higher stimulation frequencies to generate equivalent force (Fig. 1E-F), a hallmark of impaired Ca^2+^ release and excitation-contraction coupling[39]. Importantly, this defect was evident at 4–5 months, before changes in force, muscle structure, or behavior, establishing impaired Ca^2+^ handling as an early event preceding overt dysfunction (Supp. Fig. 3A). In contrast, males showed no contractile deficits, indicating that force impairment is a female-specific consequence of genotoxic stress. Both sexes displayed prolonged time to peak force and relaxation (Fig. 1F), consistent with slowed sarcoplasmic reticulum Ca^2+^ release and reuptake, identifying defective Ca^2+^ cycling as a shared consequence of ERCC1 loss[40]. However, only females progressed to impaired force production, suggesting greater susceptibility to contractile dysfunction despite a common defect in Ca^2+^ handling. Following fatigue, female SkM-KO muscles exhibited paradoxical post-fatigue force potentiation despite reduced baseline force (Fig 1F). This abnormal response is consistent with dysregulated Ca^2+^ handling and further supports impaired excitation-contraction coupling as a defining feature of the female phenotype. Males showed no comparable post-fatigue potentiation, reinforcing the sex-specific nature of the contractile defect. Collectively, these data establish a sex-dimorphic phenotypic landscape in which genotoxic stress causes functional failure without structural loss in females, and structural deterioration without functional failure in males, a dissociation that cannot be explained by a shared downstream mechanism.

### Single-nucleus transcriptomics reveals sex-specific metabolic rewiring following genotoxic stress

To define the transcriptional programs underlying the sex-dimorphic phenotypes, we performed single-nucleus RNA sequencing (snRNA-seq) of tibialis anterior (TA) muscle from male and female SkM-KO and control mice (n=3/sex/genotype; 86,641 nuclei after quality control). Cell identities were validated using canonical markers, confirming 13 distinct populations, including type IIB (Myh4), IIX (Myh1), IIA (Myh2), MuSCs (Pax7/Cd34), FAPs (Pdgfra/Dcn), endothelial cells (Pecam1/Kdr), and macrophages (Cd68/Lyz2) (Fig 3A, Supp Fig 4A)[41]. Type IIB and IIX myonuclei comprised ~74% of all nuclei, with no genotype-dependent changes in cell-type composition, indicating that ERCC1 loss reprograms transcription without altering myonuclear identity or fiber-type proportions (Fig 3B, C). Type IIA fibers exhibited the greatest transcriptional heterogeneity following ERCC1 loss, whereas macrophage programs remained largely unchanged, consistent with the absence of overt inflammation or fibrosis (Fig 3D). Notably, fast-twitch myonuclei segregated primarily by sex rather than genotype, identifying biological sex as the dominant source of transcriptional variation and establishing the need for sex-stratified analyses (Fig 3E-F).

To define functional transcriptional states in type II myonuclei, we scored curated gene sets associated with DNA repair, atrophy, and senescence. Female SkM-KO fast IIB nuclei showed a marked reduction in DNA repair pathway activity (Cliff's δ = −0.22, *p*<0.001), whereas males showed little biological change (Cliff's δ = +0.03) (Fig 3G-left). This female-specific suppression is consistent with oxidative DNA lesions impairing transcription of DNA repair genes[42-44]. Atrophy-associated signatures increased in both sexes but were significantly greater in females, paralleling their more severe functional phenotype (Fig 3G-right). In contrast, two independent senescence signatures (SenMayo and SenSig) were unchanged across genotypes (Supp. Fig. 4B), indicating that ERCC1 loss in postmitotic myofibers elicits a DNA damage response without activating canonical senescence and suggesting that the pathological consequences of genotoxic stress in myofibers operate through other molecular mechanisms rather than senescence-associated secretory programs.

To investigate sex-specific transcriptional reprogramming following DNA damage, we performed functional enrichment analysis in fast-twitch fibers (types IIB and IIX). We identified 1,316 differentially expressed genes (DEGs) among Skm-KO vs Control myofibers of both sexes, using an effect size (log(FoldChange)) and a statistical significance (adjusted P value) threshold. We then identified Molecular Signatures Database (MSigDB) pathways in which these DEGs were enriched. Functionally enriched pathways were binned into 7 categories to indicate changes in cellular transcriptional programs caused by loss of DNA-repair. Metabolism emerged as the dominant transcriptional consequence of genotoxic stress, enriched nearly threefold over all other functional categories (Fig 3H), repositioning DNA damage as a metabolic stressor rather than solely a genomic one. Rather than representing isolated pathway changes, the transcriptional response formed a coordinated metabolic cascade. In female Fast IIB myonuclei, pyruvate metabolism was driven primarily by Pdk4 and Mpc3, consistent with restricted pyruvate entry into the TCA cycle (Fig 3H-I, Supp Fig 4C-E). When pyruvate cannot be oxidized, glucose-derived carbon accumulates upstream, reflected in coordinated upregulation of glycogen synthesis genes Hk2, Ugp2, and Gys1, indicating a transcriptional shift toward glucose storage over oxidative consumption. Downstream, compensatory activation of fatty acid oxidation, phospholipid, and xenobiotic metabolism, the latter showing the largest effect size (Cliff's δ = 0.342), suggested increasing reliance on alternative fuel sources while further constraining mitochondrial capacity. At the endpoint of this cascade, purine metabolism was selectively suppressed in females (Fig 3I), indicating reduced ATP and nucleotide biosynthetic capacity, thereby limiting both energetic reserve and DNA repair potential. In contrast, males maintained or increased purine metabolism, consistent with preserved metabolic flexibility and repair capacity. Collectively, these findings identify metabolism as the principal transcriptional response to genotoxic stress and reveal fundamentally distinct metabolic adaptations between the sexes. Furthermore, female myofibers display extensive, disorganized metabolic rewiring centred on failed pyruvate oxidation and suppressed purine synthesis, while males show focused metabolic adaptation that preserves repair capacity. Lastly, unbiased driver gene analysis identified PDK4 at the origin of the female cascade, its high loading on the pyruvate metabolic score providing the mechanistic rationale for the machine learning analyses that follow (Supp Fig. 4E).

### Interpretable machine learning identifies PDK4 as a sex-biased driver of metabolic inflexibility

To identify infer transcriptional programs underlying the sex-specific transcriptional response to genotoxic stress, we applied SLIDE, an interpretable machine learning framework that infers genotype-associated transcriptional modules rather than individual differentially expressed genes[45]. SLIDE moves beyond biomarkers to the inference of a necessary and sufficient set of latent factors (in this case, context-specific co-expression modules) distinguishing outcomes of interest (the response to genotoxic stress). SLIDE comes with rigorous statistical guarantees regarding identifiability, inference and FDR control. Because SLIDE provides inference beyond prediction, these inferences can suggest possible mechanistic insights that need to be validated in downstream experiments. SLIDE has successfully helped infer novel mechanisms in different infectious and autoimmune disease contexts[46-50], as well as in cancer

Single-nucleus expression profiles were denoised using Seacells metacells before modeling using SLDIE (Fig. 4A-B). Consistent with preserved myonuclear identity (Fig. 3), control and SkM-KO metacells largely overlapped in UMAP space; however, female SkM-KO nuclei occupied distinct transcriptional states absent in controls, indicating selective transcriptional reprogramming. SLIDE robustly classified genotype within both sexes. Model performance was rigorously evaluated on held-out data (using 25 replicates of k-fold cross-validation) and significance was assessed using permutation testing (female AUC = 0.93 ± 0.01; male AUC = 0.81 ± 0.02, P < 0.01). These confirm that the inferred latent factors (context-specific co-expression modules) robustly discriminate by genotype within each sex (Fig. 4A-B; Supp Fig 5A). SLIDE resolved a transcriptional architecture in female type II myofibers centered on two dominant upstream modules (Fig. 4C). The latent factors were annotated using a LLM-based approach leveraging prior knowledge[51]. Module Z12 was enriched for fatty acid oxidation (FAO) fuel switching, whereas Module Z15 was enriched for insulin signaling and exercise-response pathways. These modules connected to (statistical interaction term in the model) downstream programs associated with substrate remodeling (Z55), myosteatosis (Z5), DLK1-DIO3 imprinting (Z29), growth factor signaling (Z42), and glucocorticoid-responsive atrogenes (Z38), indicating that the female response is organized as a coordinated regulatory network rather than independent pathway changes. Consistent with the metabolic remodeling identified in Fig 3, Z12 and Z15 defined a transcriptional trajectory linking altered substrate utilization to fiber remodeling and atrophy. Notably, the connection between insulin signaling (Z15) and glucocorticoid-responsive atrogenes (Z38) provides a transcriptional framework for the elevated atrophy signatures observed in female SkM-KO myonuclei. Investigating the discovered gene-modules further, we find Z12 and Z15 to contain the major transcriptional players that rewire the metabolism of type II fibers upon *Ercc1*-KO. These discovered genes have high predictive power (AUC) to explain the transcriptional phenotype of SkM-KO and control meta-cells (Fig 4D). Conventional univariate analyses (e.g., DEG analyses) identify simple biomarkers, while the context-specific co-expression modules identified using SLIDE provide a necessary and sufficient set of transcriptional programs that explain differences between genotypes within each sex. Further, conventional co-expression network approaches (like WGCNA) neither have guarantees on inference nor provide joint/global false discovery rate control like SLIDE.

Gene-level analyses identified PDK4 as the strongest predictor of the female genotoxic state (AUC ~0.80), followed by UCP3, whereas NR4A3 and IRS2 most strongly predicted the healthy control state (Fig 4D). SLIDE loading values likewise ranked PDK4 as the most dominant contributor (highest-loading gene) within Z12 and NR4A3 and IRS2 as the dominant contributors to Z15 (Fig 4E), identifying two mechanistically distinct but interacting regulatory programs. Correlation network analysis further established PDK4 as the central hub of Z12, exhibiting the greatest connectivity with genes involved in lipid uptake, cholesterol trafficking, and aldehyde detoxification (Fig 4F). Conversely, the Z15 network was centered on IRS2 and NR4A3, which were highly expressed in controls but suppressed in SkM-KO muscle (Fig 4G). Together, these networks define a reciprocal regulatory switch in which loss of the IRS2/NR4A3 program coincides with activation of the PDK4- centered metabolic module. In contrast, male SkM-KO myofibers exhibited a markedly different network organization (Supp Fig 5B). Although PDK4 and UCP3 were also present, they clustered with exercise-response genes including Egr1, Abra, and Nav2, rather than lipid metabolism genes, indicating an adaptive rather than pathological transcriptional context. A second male-specific module was enriched for calcium signaling genes, including Ldb2, Prkd1, Il15, and Sntb2, consistent with preserved contractile function observed in Fig 2. These findings demonstrate that PDK4 occupies fundamentally different regulatory networks in male and female myofibers, highlighting that biological sex determines its functional context following genotoxic stress.

### PDK4 mediates DNA damage-induced metabolic inflexibility and impairs myogenesis

Building on the sex-divergent metabolic signatures identified by snRNA-seq and the SLIDE model inferences, we focused on PDK4 as a candidate mediator. Single-nucleus expression profiling demonstrated that not only mean PDK4 expression but the fraction of type II myonuclei expressing PDK4 was increased in female SkM-KO relative to controls, indicating a population-level transcriptional shift rather than upregulation confined to a minority of nuclei (Fig 5A).

Western blot analysis confirmed this sex-dimorphism at the protein level with a striking inversion: male controls expressed higher baseline PDK4 than female controls (Fig 5B), consistent with the known role of PDK4 in male fasting-induced metabolic adaptation, yet ERCC1 loss drove a significant PDK4 increase in females and a significant decrease in males, reversing the baseline sex relationship entirely (Fig 5C). Given that PDK4 inhibits the pyruvate dehydrogenase complex (PDC) via phosphorylation, thereby supressing glucose oxidation (Fig. 5D), we assessed phosphorylated PDH (p-PDH(Ser293)) as a direct functional readout of PDK4-mediated PDC inhibition. Female SkM-KO mice showed a significant elevation in p-PDH compared to controls (Supp Fig 6A), confirming enhanced PDK4-mediated suppression of glucose oxidation and indicating a shift towards reduced metabolic flexibility.

Liquid chromatography-mass spectrometry (LC-MS) metabolomics directly validated the predicted metabolic divergence. Female SkM-KO muscles exhibited elevated lactate, a trend toward pyruvate accumulation, and increased CoASH, consistent with PDK4-mediated inhibition of pyruvate dehydrogenase (PDC), reduced pyruvate oxidation, and diversion toward lactate production (Fig. 5E-F). In contrast, males showed no lactate accumulation, significantly increased acetyl-CoA, and only a modest increase in CoASH, indicating preserved pyruvate flux into the TCA cycle (Supp Fig. 6B). Together, these metabolite profiles confirm that ERCC1 loss drives fundamentally distinct metabolic states: PDK4-dependent pyruvate-to-lactate shunting in females versus preserved oxidative metabolism in males, providing direct biochemical validation of the SLIDE predictions. Despite these divergent metabolic outcomes, both sexes exhibited elevated CoASSG, indicating that oxidative stress is a shared consequence of genotoxic DNA damage (Fig 5F, Supp Fig. 6C). Thus, the sex-dimorphic phenotype arises not from differences in the initial oxidative insult, but from fundamentally different downstream metabolic responses.

To establish PDK4 as a causal mediator of genotoxic metabolic dysfunction, we used female-derived C2C12 myoblasts differentiated in the presence of doxorubicin as an in vitro model of genotoxic stress. DNA damage was confirmed by robust γH2AX induction two hours after doxorubicin exposure, validating the genotoxic model prior to all downstream analyses (Supp Fig 6D). Consistent with *in vivo* findings, doxorubicin increased PDK4 expression and p-PDH (Fig 5G and Supp Fig 6E), corroborating PDK4-mediated PDC inhibition in the genotoxin-damaged myotube system. To characterize the metabolic consequences of PDK4 activation, we measured fatty acid oxidation rate directly using ^14^C-oleate oxidation. Doxorubicin significantly increased fatty acid oxidation (Fig 5H), confirming the substrate switch from glucose oxidation to lipid utilization predicted by the snRNA-seq FAO fuel-switch signature. This metabolic shift was accompanied by impaired myogenic differentiation, with doxorubicin-treated cells forming significantly fewer multinucleated eMHC-positive myotubes (Fig 5N). The *in vitro* C2C12 data establish PDK4-driven metabolic dysfunction at the cellular level, but cannot resolve whether this metabolic failure translates to impaired Ca^2+^ handling and contractile dysfunction at tissue scale, the functional deficits that define the clinical frailty phenotype. To address this, we used three-dimensional (3D) bioengineered skeletal muscle constructs with simultaneous real-time Ca^2+^ imaging and contractile measurements. Doxorubicin-treated constructs exhibited marked functional impairment, with ~50% lower Ca^2+^ transient amplitude (*p*<0.001), ~70% reduced contractile distance (*p*<0.0001), and significantly shortened 10–90% rise time, indicating disrupted excitation-contraction coupling (Fig 5I-L). In contrast, fiber width, branching, and alignment remained unchanged, demonstrating that these deficits arise from metabolic dysfunction rather than structural deterioration. Together, these findings show that PDK4-driven metabolic dysfunction is sufficient to impair tissue-scale Ca^2+^ signalling and contractile function, providing a mechanistic explanation for the contractile deficits observed in female SkM-KO muscle and recapitulating the structural-functional uncoupling observed *in vivo*.

Pharmacological PDK4 inhibition reduced p-PDH to near-control levels (Fig. 5M), directly confirming that PDK4 kinase activity is responsible for the observed PDH phosphorylation rather than a PDK4-independent stress response. Notably, PDK4 inhibition significantly restored myotube formation, implicating PDK4 as a mediator of DNA damage-induced defects in myogenesis (Fig. 5N-O). Because PDK4-mediated inhibition of PDH is expected to restrict pyruvate entry into the TCA cycle, we next examined whether accumulated pyruvate was diverted toward lactate production. Doxorubicin increased extracellular lactate levels, whereas PDK4 inhibition reduced lactate accumulation (Supp Fig 6F). Doxorubicin increased LDHA expression, consistent with diversion of pyruvate toward lactate, which was reversed by PDK4 inhibition (Fig. 5P). Doxorubicin also reduced oxygen consumption rate (OCR) during myogenic differentiation, whereas PDK4 inhibition restored OCR to control levels (Fig. 5Q). Together, these findings demonstrate that PDK4-mediated PDC inhibition redirects pyruvate away from mitochondrial oxidation and that blocking PDK4 restores pyruvate flux through the TCA cycle and mitochondrial function in genotoxin-damaged myotubes. To establish that the PDK4-dependent metabolic failure is not specific to doxorubicin, we exposed myotubes to cisplatin and 5-fluorouracil, genotoxins with distinct mechanisms of DNA damage. Both treatments markedly impaired myotube formation compared with untreated controls, whereas PDK4 inhibition significantly rescued myogenic differentiation in each condition (Supp Fig. 6G). This generalizability across three chemotherapeutic agents with different lesion spectra confirms that the PDK4 axis responds to genotoxic stress broadly rather than to doxorubicin-specific off-target effects, directly supporting the translational relevance of the mechanism to the diverse genotoxic exposures, environmental toxicants, radiation, and chemotherapy, that affect skeletal muscle in humans. Collectively, these data establish PDK4 as causally required for DNA damage-induced metabolic inflexibility that diverts pyruvate away from the TCA cycle, depleting TCA intermediates and collapsing mitochondrial oxidative capacity: the biochemical bottleneck that pharmacological PDK4 inhibition directly relieves.

### α-Ketoglutarate bypasses the PDK4 metabolic bottleneck to restore muscle function

Having established PDK4 as the driver of TCA cycle disruption in female genotoxic muscle, we asked whether this metabolic bottleneck creates a therapeutically targetable vulnerability. High-resolution respirometry of isolated TA myofibers revealed a striking sex difference: female SkM-KO fibers significantly increased oxygen consumption in response to glutamate, but not palmitate, indicating preferential reliance on glutamate as an alternative anaplerotic fuel source (Fig. 6A). This glutamate dependence is consistent with PDK4-mediated inhibition of PDC: as pyruvate entry into the TCA cycle is restricted and TCA intermediates become depleted, myofibers increasingly rely on glutamate conversion to α-ketoglutarate to replenish the cycle downstream of the metabolic block (Fig 6B). In contrast, male SkM-KO fibers showed reduced oxygen consumption following glutamate addition (Supp. Fig. 7A), consistent with preserved pyruvate oxidation and metabolic flexibility.

Although α-ketoglutarate (αKG) has previously been studied as a broad aging intervention through mechanisms such as TOR inhibition, ATP synthase modulation, and epigenetic regulation, our therapeutic strategy is mechanistically distinct. Rather than using αKG as a pleiotropic longevity supplement, we employed it as a targeted anaplerotic therapy to bypass the PDK4-induced TCA bottleneck identified by our metabolomic and respirometry analyses. Second, the intervention exploits a previously unrecognized female-specific anaplerotic vulnerability created by PDK4 induction, a metabolic dependency not present in males. Third, the mechanism of action, direct TCA intermediate replenishment bypassing the enzymatic block, is orthogonal to all previously proposed αKG mechanisms, none of which involve TCA anaplerosis in a PDK4-dependent context. The rationale for αKG supplementation arose directly from the experimental observation that female genotoxic muscle preferentially relies on glutamate-derived αKG to sustain oxidative metabolism. To test whether replenishing the TCA cycle downstream of the PDK4 block could prevent metabolic dysfunction, female SkM-KO mice were supplemented with dietary calcium αKG beginning at 6 months of age, before overt phenotypic decline.

Dietary αKG supplementation produced comprehensive functional rescue in female SkM-KO mice across every endpoint assessed. Motor coordination and balance were significantly improved, as measured by rotarod performance (Fig. 6C). Direct contractile assessment of the TA revealed enhanced force production and improved stimulation frequency response- the latter reflecting restoration of Ca^2+^ transient summation efficiency that was identified as the earliest functional deficit in young SkM-KO females (Fig. 6D-E). Furthermore, temporal contractile properties were significantly improved, with reductions in time to maximum contractile force and half-relaxation time, indicating restored Ca^2+^ cycling kinetics consistent with SERCA reactivation downstream of ATP recovery (Fig. 6F-G). Fatigue recovery was also improved, further confirming restoration of the energetic infrastructure required for sustained contractile function (Fig. 6H). Critically, these improvements occurred despite persistent ERCC1 deficiency, ongoing DNA damage, and sustained PDK4 induction, demonstrating that restoring TCA intermediate availability is sufficient to rescue muscle function downstream of the genetic defect.

Accordingly, metabolomic profiling of glycolytic and TCA cycle intermediates in α-KG-fed SkM-KO muscle revealed no significant differences in pyruvate and lactate levels compared with controls (Fig. 6I), in contrast to the metabolic alterations observed in untreated SkM-KO muscle (Fig. 5E). These findings suggest that α-KG supplementation counteracts the metabolic consequences of PDK4-mediated PDH inhibition, thereby restoring carbon flux into the TCA cycle. Likewise, the elevated CoASH levels observed in untreated SkM-KO muscle (Fig. 5F) were no longer detected following α-KG supplementation, indicating improved utilization of free coenzyme A within the TCA cycle (Fig. 6I). Furthermore, CoASSG levels were also normalized (Fig. 6I), in contrast to the increased levels observed in untreated SkM-KO muscle (Fig. 5F), consistent with the restoration of redox homeostasis by the α-KG diet. The functional rescue was accompanied by biochemical evidence that αKG directly relieves the PDK4-induced TCA bottleneck.

To determine whether α-KG supplementation alleviates lactate dysregulation downstream of PDK4 activation, we assessed LDHA expression in TA muscle and serum lactate levels. Untreated SkM-KO mice exhibited significantly increased LDHA expression in TA muscle, accompanied by reduced lactate levels in the serum (Fig. 6J-K), indicating intramuscular lactate accumulation driven by increased production and impaired MCT- mediated export from energetically compromised myofibers. In contrast, α-KG supplementation partially reduced LDHA expression and was associated with a partial restoration of serum lactate levels (Fig. 6J-K), suggesting that TCA replenishment reduces lactate production while restoring the energetic capacity required for efficient lactate export. The partial normalization of lactate handling is consistent with αKG acting downstream of PDK4, restoring TCA metabolism without reversing PDK4-mediated PDH inhibition. We next examined the expression of p21 and Atrogin, key markers of DNA damage-induced cell cycle arrest and muscle atrophy, respectively. Untreated SkM-KO muscle exhibited elevated p21 and Atrogin-1 expression compared with controls. αKG supplementation significantly reduced Atrogin-1 without affecting p21 (Supp Fig. 7B), indicating that TCA replenishment suppresses the downstream atrophy program while leaving the DNA damage response intact. These findings demonstrate that αKG rescues muscle function by bypassing the metabolic consequences of genotoxic stress rather than reducing DNA damage itself.

To confirm that αKG acts directly on the myofiber rather than through systemic or endocrine effects, and establish that αKG directly rescues genotoxic myofibers downstream of the PDK4 block, we supplemented doxorubicin-treated C2C12 myotubes with αKG. α-KG supplementation in the presence of doxorubicin-induced DNA damage significantly increased myotube formation (Fig. 6L, Supp Fig. 7C), an effect comparable in magnitude to that achieved by PDK4 inhibition (Fig. 5J-K). To determine whether αKG and PDK4 inhibition act through distinct or convergent mechanisms, we treated doxorubicin-damaged C2C12 myotubes with both interventions simultaneously. Combined treatment rescued myogenic differentiation to the same extent as either intervention alone, with no additive or synergistic benefit (Fig 6L, Supp Fig 7C). This finding indicates that αKG and PDK4 inhibition converge on the same rate-limiting metabolic bottleneck: PDK4 inhibition restores pyruvate entry into the TCA cycle upstream, whereas αKG replenishes TCA intermediates downstream. The lack of additional rescue with combination treatment demonstrates that TCA intermediate availability is the primary limiting factor for myogenic recovery under genotoxic stress, and that restoring this bottleneck by either approach is sufficient for maximal functional rescue.

To determine whether TCA replenishment restores functional muscle properties beyond myogenic differentiation, we tested αKG in C2C12 3D constructs with simultaneous Ca^2+^ imaging and force measurements. This platform uniquely combines cellular-resolution Ca^2+^ imaging with preserved tissue-level excitation-contraction coupling. αKG significantly rescued two of the three functional deficits induced by doxorubicin: Ca^2+^ transient amplitude was partially restored (*p*<0.05), and contractile distance, the most severely impaired parameter, was markedly improved (*p*<0.01), indicating recovery of excitation-contraction coupling and force-generating capacity. In contrast, 10–90% rise time was not significantly restored, suggesting that TCA replenishment preferentially rescues energetic deficits underlying force generation while not fully correcting all aspects of Ca^2+^ kinetics (Fig. 6M-O), Supp Fig. 7D). The partial recovery of Ca^2+^ amplitude is consistent with αKG acting downstream of the PDK4 block, replenishing TCA intermediates and restoring mitochondrial ATP production without reversing PDK4-mediated inhibition of pyruvate oxidation. Together, these findings demonstrate that bypassing the PDK4-induced metabolic bottleneck is sufficient to restore the primary functional consequence of genotoxic stress- contractile performance- establishing αKG as a mechanistically targeted intervention for genotoxic muscle dysfunction.

Collectively, these findings establish that αKG rescues female genotoxic muscle dysfunction through a sex-specific, PDK4-dependent mechanism. Across four complementary systems- metabolomics, *in vivo* functional studies, female-derived C2C12 myotubes, and bioengineered human skeletal muscle constructs- αKG replenished TCA intermediates downstream of the PDK4 block, restoring mitochondrial metabolism, normalizing lactate and CoA homeostasis, improving myogenic differentiation, recovering muscle force and Ca^2+^ handling, and rescuing tissue-level excitation-contraction coupling. Importantly, these benefits occurred despite persistent ERCC1 deficiency, DNA damage, and PDK4 induction, demonstrating that bypassing the metabolic bottleneck is sufficient to restore muscle function. The consistent rescue observed in females, where PDK4 drives TCA depletion, confirms that αKG acts as a targeted metabolic bypass rather than a general anti-aging intervention, providing a mechanistically defined and translationally relevant therapeutic strategy for female genotoxic muscle dysfunction.

### PDK4 defines a conserved metabolic axis in aging human type II myofibers

To investigate whether the molecular signatures of DNA-damage induced atrophy in mice translate to changes that occur in the human skeletal muscle with natural aging, we aimed to analyse a comprehensive transcriptomic cell atlas of the human locomotor skeletal muscle across different sexes and ages, including individuals aged ≥84 years with signs of sarcopenia [52]. To assess the transcriptional changes that female type II myofibers undergo with ageing, we picked a young age-group (1 human donor, 2 replicates, age = 34) and an old age-group (2 human donors, 4 replicates each, age = 80) with overall 13,064 single-cells (Fig 7A). Although the young cohort comprised a single donor with two replicates, our analysis focused on within-sample pseudotime trajectories rather than cross-donor comparisons, minimizing the impact of donor variability. The concordance between pseudotime- and chronological age-based analyses provides internal validation that the observed transcriptional changes reflect aging rather than donor-specific effects. Post QC to retain type II myofiber nuclei with high DNA-damage burden, we obtained a total of 3,989 single-cells, activity scores of which show a marked increase in DNA-Damage response (DDR) with chronological age, supporting our DNA-damaged *Ercc1*-knockout mouse models (Cliff’s delta = 0.263 displaying a consistent upregulation of the pathway in the 80-year-olds) (Fig 7B). As multiple environment and lifestyle factors can influence natural human aging, we also performed the analysis across pseudotime to account for transcriptional similarity in placing the cells across a trajectory of natural aging. Pseudotime correlated significantly with chronological age (R^2^ = 0.102, *p* = 4.75 × 10^−89^), demonstrating that the inferred trajectory captures biological aging rather than technical variation. Although the effect size was modest, this is expected because transcriptional state is influenced by factors beyond chronological age, including lifestyle, environmental exposures, and inter-individual variability (Supp Fig 8A). Furthermore, DNA-damage response pathway shows an increase in activity across pseudotime (Supp Fig 8B), consistent with the trend observed with natural chronological age. Interestingly, we also see an activation of *PDK4* transcription with chronological age (Fig 7C), and along pseudotime aging trajectory (Supp Fig 8B) in human females. This discovery of increased *PDK4* in human females, led us to perform cross-species analysis of the transferability of learnt gene-modules from female *Ercc1*-KO mice to explain the chronological age differences in female humans. Machine learning model weights of the gene module Z12 (Figure 4E) learnt from female mice meta-cells were used to calculate an activity score of that pathway (FAO Fuel Switch) in human cells. The mouse-derived Z12 gene module predicted human aging with an AUC of 0.629, despite being transferred directly to the human dataset without retraining or fine-tuning. This above-chance performance is notable given the differences in species, tissue processing, and the underlying biology of ERCC1 deficiency in mice versus naturally accumulated DNA damage in aging humans. Consistent with this, the pathway activity score showed strong separation between groups (Cliff's δ = 0.258), indicating that although the module does not fully predict chronological age- which is influenced by many non-transcriptional factors- the Z12 fatty acid oxidation fuel-switch program is robustly conserved between female genotoxic mouse myofibers and aged human type II myonuclei with elevated DNA damage (Figure 7D-E). Driver genes for these scores indicate conserved transcriptional signatures of metabolic rewiring between female mice upon *Ercc1* deletion and female human cells containing high DNA-damage burden in type II fibers. We also assessed the changes in literature-derived pathways for these human samples and observed consistent metabolic reprogramming of upregulated Pyruvate metabolism, Fatty Acid Oxidation and downregulation of Glycogen, indicating that in humans too with natural aging, type II fibers rely on fatty acid metabolism through upregulating *PDK4* (Figure 7F-G, Supp Fig 8C).

To investigate the causality of this claim, we utilized an *in-silico* dynamic perturbation Large Language Model (LLM) pretrained on 175M+ human cells across natural aging pseudotime trajectories ranging from first to tenth decade of lifespan [52]. *In silico* PDK4 inhibition across 2,000 aged human type II myonuclei produced a significant negative Δt, shifting cells toward younger pseudotime states and indicating that PDK4 actively drives the aged transcriptional program rather than simply correlating with it. Perturbations across the PDK4–IRS2–NR4A3 axis were highly consistent with the mouse data: PDK4 inhibition was rejuvenating, whereas PDK4 overexpression accelerated transcriptional aging (Fig 7H, Supp Fig 8D). IRS2 inhibition produced the strongest pro-aging effect of any perturbation, consistent with its role as the upstream brake on the PDK4 program, while NR4A3 inhibition was also pro-aging. In contrast, overexpression of IRS2 or NR4A3 produced little or no rejuvenation, indicating that restoring upstream regulators is insufficient once the PDK4-driven metabolic program is established. Together, these findings identify PDK4 inhibition as the most effective therapeutic entry point for reversing the aged transcriptional program.

To determine whether aging is associated with impaired myogenic differentiation and activation of the PDK4–PDH metabolic axis in human skeletal muscle, we first compared the differentiation capacity of primary skeletal muscle cells derived from young and aged female donors. Cells from young donors underwent robust myogenic differentiation, with differentiated myotubes readily apparent within 48 h. In contrast, cells from aged donors showed a pronounced impairment in differentiation, with little to no evident myotube formation over the same period, indicating an age-associated loss of myogenic capacity (Fig. 7I). Next, to access PDK4 induction in aging human muscle results in functional suppression of pyruvate oxidation, we measured PDK4 protein and PDH phosphorylation in primary skeletal muscle cells from young and aged female donors during myogenic differentiation (48, 72, and 96 h). This differentiation model recapitulates the transition to the postmitotic state, where persistent DNA damage accumulates and the PDK4-driven metabolic program was defined in the SkM-KO model. PDK4 expression was significantly higher in aged cells at all time points and was accompanied by increased PDH phosphorylation (Fig 7J), demonstrating that age-associated PDK4 induction translates into active inhibition of pyruvate dehydrogenase rather than a transcriptional change alone. Importantly, both PDK4 expression and PDH phosphorylation progressively increased during differentiation in aged, but not young, cells, indicating that the metabolic block intensifies as myofibers become postmitotic. This temporal pattern mirrors the progressive functional decline observed in female SkM-KO mice and complements the human snRNA-seq and in silico perturbation analyses, completing the translational framework: the PDK4–PDH axis identified as the driver of female genotoxic muscle dysfunction in mice is conserved at the transcriptional, enzymatic, and functional levels in aging human skeletal muscle. Collectively, these findings demonstrate that the PDK4-centered metabolic program identified in female genotoxic mouse muscle is conserved in naturally aging human female type II myofibers.

## DISCUSSION

DNA damage has largely been viewed as a monolithic hallmark of aging whose effects scale with lesion burden. Our findings challenge this paradigm in postmitotic skeletal muscle by showing that the same genotoxic stress generates fundamentally different DNA lesion landscapes in male and female type II myofibers- oxidative lesions in females and uracil misincorporation in males. These distinct lesion classes engage divergent transcriptional and metabolic programs that determine whether myofibers preserve contractile function or progress to metabolic failure. Together, these findings redefine sex-specific muscle aging as a cell-intrinsic consequence of DNA lesion chemistry rather than simply differences in damage burden.

A critical and largely overlooked aspect of skeletal muscle aging is the cellular context in which DNA damage accumulates. Most studies have focused on proliferating satellite cells and myoblasts, linking genotoxic stress to stem cell exhaustion, impaired regeneration, and reduced myogenic potential[53-57]. While these mechanisms are important, they do not directly address the cells responsible for muscle force generation. In contrast, skeletal muscle is composed predominantly of postmitotic type II myofibers, which cannot dilute DNA lesions through cell division or replace damaged nuclei through proliferation. Consequently, DNA damage persists, chronically disrupting transcription and progressively remodelling cellular metabolism[56, 58, 59]. To our knowledge, this is the first study to define the consequences of DNA damage accumulation specifically in postmitotic fast-twitch type II myofibers, the fiber population most vulnerable to aging and most strongly associated with functional decline. This distinction is biologically significant because postmitotic myonuclei possess lower DNA repair capacity than proliferating myogenic cells, making identical lesions likely to produce fundamentally different transcriptional and metabolic outcomes. The sex-specific lesion landscapes, metabolic reprogramming, and contractile dysfunction identified here therefore arise from the postmitotic myofiber nucleus itself, providing a mechanistic framework for understanding why muscle weakness and metabolic dysfunction emerge before overt depletion of the satellite cell pool.

A key question raised by our findings is why identical ERCC1 loss generates oxidative lesions in females but uracil misincorporation in males. We propose two non-mutually exclusive mechanisms. First, female type II myofibers may experience greater oxidative stress through sex-specific differences in mitochondrial ROS production, extracellular matrix-derived H_2_O_2_, or reduced antioxidant defenses, preferentially generating 8-oxoG lesions. Second, male myofibers may preferentially accumulate uracil through APOBEC cytidine deaminase activity[60-62], consistent with the higher basal UDG signal observed in males. Distinguishing between these mechanisms will require direct measurements of ROS production, deaminase activity, and chromatin accessibility in sex-stratified primary myofibers. Regardless of the upstream source, published evidence establishes that oxidative lesions impede RNA polymerase II whereas uracil misincorporation does not, providing a plausible lesion-specific mechanism that could contribute to the divergent transcriptional responses observed between sexes, though a direct causal link between lesion type and PDK4 induction remains to be experimentally established in myofibers [44, 63-65].

The relative functional preservation of male SkM-KO muscle should not be interpreted as protection from pathology. Instead, males and females appear to follow distinct pathological trajectories under the same genotoxic stress: females develop metabolic dysfunction and contractile failure with minimal structural change, whereas males exhibit fiber loss, myosteatosis, and tissue remodelling while largely preserving function within the 8–9-month period studied. Whether males ultimately develop functional decline through a PDK4-independent mechanism remains an important question. The selective loss of type IIB fibers and lean-to-fat tissue replacement in male muscle suggest a progressive remodelling program that may compromise function at later stages. Longitudinal studies extending into advanced age will determine whether PDK4 suppression provides sustained protection or simply delays the onset of genotoxic muscle dysfunction in males.

Although αKG has been investigated as a broad aging intervention through mechanisms including TOR inhibition, ATP synthase modulation, and epigenetic regulation[66-70], the mechanism identified here is fundamentally different. Rather than acting as a pleiotropic longevity supplement, αKG restores muscle function by replenishing TCA intermediates downstream of a defined PDK4-driven metabolic bottleneck. Four features distinguish this mechanism from prior αKG studies. First, it is cell-type specific, operating in postmitotic type II myofibers undergoing genotoxic metabolic reprogramming. Second, it is sex-specific, targeting an anaplerotic vulnerability created by PDK4 induction in females but absent in males. Third, αKG acts through direct TCA anaplerosis, bypassing the PDK4-mediated block rather than modulating canonical aging pathways. Finally, its therapeutic rationale emerges directly from our experimental data, with female-specific glutamate dependence identifying αKG as the optimal downstream metabolic substrate. Notably, PDK4 inhibition and αKG supplementation produced equivalent, non-additive rescue, demonstrating that both interventions converge on the same rate-limiting metabolic bottleneck and suggesting TCA intermediate depletion as the possible primary driver of genotoxic muscle dysfunction. These findings carry direct translational relevance. PDK inhibitors, including dichloroacetate, have an established pharmacological safety record from clinical use in lactic acidosis and mitochondrial disease, while dietary α-ketoglutarate is already available as an accessible, well-tolerated supplement, positioning both arms of this mechanism for possible translation into at-risk populations. Female cancer survivors exposed to genotoxic chemotherapy, who exhibit premature frailty at nearly three times the rate of male survivors, represent a mechanistically aligned and clinically identifiable population in which adjunctive PDK4 inhibition or α-ketoglutarate supplementation represents a mechanistically motivated candidate strategy that warrants prospective evaluation in this population.

The 3D muscle construct studies further refine the mechanism of αKG rescue. αKG markedly improved contractile distance, the most severely impaired functional parameter, and partially restored Ca^2+^ transient amplitude, but did not normalize 10–90% rise time. This dissociation suggests that TCA replenishment preferentially restores the energetic capacity required for effective Ca^2+^ release and force generation, while other aspects of Ca^2+^ kinetics remain impaired. The persistent alteration in rise time is consistent with residual defects in the Ca^2+^ release machinery that are not fully corrected by restoring metabolic flux alone. These findings suggest that combining TCA replenishment with interventions targeting Ca^2+^ handling may provide more complete functional rescue than either strategy alone, an important avenue for future investigation.

The paradoxical post-fatigue force potentiation observed in female SkM-KO muscle, where force recovered to nearly twice baseline despite reduced tetanic force, is consistent with the PDK4-lactate model. Elevated intramuscular lactate resulting from PDK4-driven pyruvate diversion may transiently sensitize RyR1 to Ca^2+^-induced Ca^2+^ release, enhancing sarcoplasmic reticulum Ca^2+^ release during post-fatigue recovery despite reduced baseline Ca^2+^ stores. This interpretation is consistent with the elevated lactate levels observed in female SkM-KO muscle, though it remains speculative and direct evidence linking PDK4-driven lactate accumulation to RyR1 sensitization in this model has not been established.

The identification of IRS2 and NR4A3 as the dominant hubs of the Z15 insulin signalling and exercise-response module provides a mechanistic explanation for the sustained PDK4 induction observed in female myofibers. As central regulators of insulin signaling and oxidative metabolism, IRS2 and NR4A3 normally suppress PDK4 expression [71-74]. Their coordinated downregulation in female SkM-KO myonuclei, identified by SLIDE and confirmed by per-gene AUC analysis, is consistent with reduced suppression of PDK4 expression, though direct experimental restoration of IRS2 or NR4A3 to confirm this regulatory switch has not yet been performed and represents an important priority for future work. Computational in silico perturbation of human aging data suggests that this regulatory architecture may be relevant to natural aging: IRS2 inhibition produced the strongest pro-aging transcriptional shift of any perturbation tested, raising the possibility that IRS2 loss is an early event in the human aging trajectory, though these analyses are computational predictions and have not been validated by functional experiments in aged human tissue. In contrast, overexpression of IRS2 or NR4A3 failed to rejuvenate aged myonuclei, indicating that once the PDK4-driven metabolic program is established, restoring upstream regulators is insufficient to reverse it. These findings suggest that PDK4 inhibition or downstream TCA replenishment may represent more tractable intervention strategies than upstream regulator restoration.

In conclusion, our findings establish a new principle in the biology of aging: in postmitotic type II myofibers, the qualitative nature of DNA damage, shaped by biological sex, is associated with divergent metabolic fates. Oxidative lesions accumulating in females are associated with a PDK4-centered metabolic program, driving metabolic inflexibility, TCA intermediate depletion, impaired Ca^2+^ handling, and contractile dysfunction that precedes overt structural deterioration. Males accumulate transcriptionally permissive uracil lesions, preserving metabolic flexibility and muscle function despite greater structural remodeling within the timeframe studied. By replenishing TCA intermediates downstream of the PDK4 block, αKG rescues this metabolic defect across complementary *in vivo*, cellular, tissue-engineered, and human systems, establishing it as a mechanistically targeted metabolic bypass rather than a general anti-aging intervention. In naturally aging human female type II myofibers, preliminary evidence suggests conservation of PDK4 upregulation and associated metabolic signatures, and computational modeling indicates that in silico PDK4 inhibition shifts aged myonuclei toward a more youthful transcriptional state, though functional validation of PDK4 inhibition in aged human muscle tissue was not performed in the current study. More broadly, our data raise the possibility that DNA lesion chemistry, rather than simply lesion burden, may be an important determinant of sex-specific aging trajectories in postmitotic tissues, providing a framework that warrants systematic investigation across tissue types and in human populations.

## Methods

### Ethical Approval

All animal experiments were approved by the University of Pittsburgh’s Institutional Animal Care and Use Committee. These experiments were conducted in accordance with protocol 24075273. Experiments were performed using 4 month and 9 month old male and female ERCC1 KO mice. All mice were obtained from Jackson Laboratories.

### Generation of skeletal muscle-specific *Ercc1* knockout mice

A conditional *Ercc1* allele was generated using CRISPR–Cas9-mediated genome editing. Exon 5 was selected as the critical exon and flanked by loxP sites such that Cre-mediated excision results in splicing of exon 4 to exon 6, producing a frameshift and premature termination codon. CRISPR guide RNAs targeting the intronic regions flanking exon 5 were designed using CRISPOR and evaluated for predicted activity and potential off-target sites. Following assessment of editing efficiency in mouse embryos, the Ercc1-i5-1 and Ercc1-i6-1 guide RNAs were selected for generation of the floxed allele. A long single-stranded DNA donor (Ercc1-flox-ssODN; Megamer, Integrated DNA Technologies) containing two loxP sites and diagnostic EcoRI restriction sites was used as the homology-directed repair template, with 112-bp and 150-bp 5′ and 3′ homology arms, respectively.

Fertilized C57BL/6J embryos generated by natural mating were pronuclear microinjected with 0.33 μM EnGen Cas9 protein (New England Biolabs, M0646T), Ercc1-i5-1 and Ercc1-i6-1 sgRNAs (21.23 ng μl^−1^ each) and Ercc1-flox-ssODN (10 ng μl^−1^). Injected embryos were cultured overnight, and embryos that reached the two-cell stage were transferred into the oviducts of pseudopregnant CD1 recipient females.

Founder mice were screened by PCR using the Ercc1-i5-F2 (5′-CTGTATTTCTGCGTCTGAGGGA-3′) and Ercc1-i6-R1 (5′-CAATGAGCCATGCCCCCA-3′) primers with Q5 High-Fidelity DNA Polymerase (New England Biolabs). PCR products were subjected to EcoRI restriction fragment length polymorphism analysis to detect incorporation of the diagnostic EcoRI sites adjacent to the loxP sequences. Candidate alleles were further validated by gel purification, TOPO cloning and sequencing, and sequence reads were aligned to the C57BL/6J reference genome (GRCm38/mm10) to confirm correct incorporation of the floxed allele. Correctly targeted founders were bred with wild-type C57BL/6J mice to establish germline transmission, and heterozygous offspring were subsequently intercrossed to generate homozygous *Ercc1*fl/fl mice.

For skeletal muscle-specific deletion of *Ercc1, Ercc1*fl/fl mice were crossed with Myl1-Cre mice (STOCK Myl1tm1(cre)Sjb/J; The Jackson Laboratory, stock no. 024713; RRID: IMSR_JAX:024713). This Cre allele expresses Cre recombinase under control of the *Myl1* locus and has been established for selective Cre-mediated recombination in skeletal muscle fibers. Skeletal muscle-specific *Ercc1* knockout mice (*Ercc1*fl/fl;Myl1-Cre+) were generated by combining the homozygous floxed *Ercc1* allele with the Myl1-Cre allele. Cre-negative *Ercc1*fl/fl littermates were used as controls. All Experimental mice were housed in a pathogen-free animal facility at the Center for Biotechnology and Bioengineering, University of Pittsburgh and maintained in a 12 h light/dark cycle, at 20–22 °C temperature and 50–65% air humidity.

### Single-cell preparation and extraction from skeletal muscle

Single Nuclei were isolated from frozen TA tissue using the Chromium Nuclei Isolation Kit (10x Genomics) according to the manufacturer’s instructions. All procedures were performed on ice, and centrifugation steps were conducted at 4 °C. Frozen tissue (3–50 mg) was mechanically dissociated in 200 μl Lysis Buffer, followed by addition of a further 300 μl Lysis Buffer and incubation on ice for 10 min. The homogenate was passed through a pre-chilled Nuclei Isolation Column by centrifugation at 16,000g for 20 s. The flow-through was vortexed and centrifuged at 500g for 3 min. The nuclear pellet was resuspended in 500 μl Debris Removal Buffer and centrifuged at 700g for 10 min. Nuclei were subsequently washed twice in 1 ml Wash and Resuspension Buffer by centrifugation at 500g for 5 min. The final pellet was resuspended in 50–500 μl Wash and Resuspension Buffer, depending on nuclei yield. Nuclei concentration and integrity were assessed using propidium iodide staining, and samples were maintained on ice until immediate loading onto the 10x Genomics platform.

### snRNA-seq raw data processing, clustering and cell type annotation

#### 1.1 Raw data processing with CellBender

Ambient (background) RNA was removed from the raw CellRanger count matrices with CellBender remove-background (a deep generative model of empty-droplet contamination). Male and female samples were processed separately, yielding CellBender-corrected count matrices that were retained as a dedicated cellbender layer alongside the raw CellRanger counts. CellBender per-droplet estimates (background fraction, cell probability, cell size, droplet efficiency) and the run-level learning-curve and target-false-positive-rate parameters were carried forward as metadata. Downstream quality control was performed in Scanpy. Per-nucleus QC metrics were computed with sc.pp.calculate_qc_metrics using mitochondrial (mt-), ribosomal (rps/rpl) and haemoglobin (hb) gene classes. Nuclei were retained when the CellBender posterior cell probability exceeded 0.99, total mitochondrial counts were below the comparability threshold (query samples, <300; reference samples, <50), and at least 200 genes were detected (sc.pp.filter_cells, min_genes=200). Predicted doublets (Scrublet) were removed, and duplicate barcodes arising from pool merging were collapsed to their first occurrence. Genes detected in fewer than 3 nuclei or with fewer than 10 total counts were discarded. After filtering, the query atlas (mice from 2025 batch) comprised 50,394 nuclei (26,564 male; 23,918 female).

#### 1.2 Batch integration, clustering and cell-type annotation

The query nuclei (2025 batch/cohort) were integrated with a reference atlas (2024 batch/cohort) using scvi-tools (v1.4.0). The two datasets were concatenated on their common genes, leading to a total feature space of 20,384 genes (mitochondrial, ribosomal and haemoglobin genes were excluded). Counts were normalised to a fixed library size and log-transformed (sc.pp.normalize_total, target_sum=1e4) for visualization, with the full-dimension object stored in raw-counts layer. Highly variable genes were selected with the seurat_v3 flavour (n_top_genes=3000) on the raw-count layer, by batch. A variational autoencoder (scvi.model.SCVI; n_layers=2, n_latent=32, n_hidden=128) was trained with the cohort as batch key, producing a 32-dimensional latent embedding (X_scVI). A neighbourhood graph and UMAP (min_dist=0.3) were computed on this embedding. Cell-type labels were transferred from the reference to the query with scvi.model.SCANVI.from_scvi_model, initialized from the trained scVI model and trained for 20 epochs with 100 cells per label; query nuclei were predicted (C_scANVI) and a joint X_scANVI embedding recomputed. Reference labels were originally defined in Seurat (v5) by canonical marker-gene panels. Cluster-specific markers were computed for each louvain cluster with FindAllMarkers (Wilcoxon rank-sum, one-versus-rest, only.pos = FALSE), retaining genes with avg_log2FC > 1, with per-cluster discriminative power cross-checked using FindMarkers (test.use = "roc"). Each cluster was then matched to a cell type by intersecting its top-ranked markers against the canonical panel curated from Perez et al.[75] and re-labeled with RenameIdents. This resolved 13 cell-type populations across the integrated atlas. Marker genes per population were confirmed with sc.tl.rank_genes_groups (Wilcoxon rank-sum test) in the integrated dataset across both batches/cohort. On the combined query + reference object, residual batch effects were corrected with Harmony (sc.external.pp.harmony_integrate, harmonypy python library) on the PCA embedding, using batch (cohort) as the correction key with theta = 4 and max_iter_harmony = 100 (lamb = 1, sigma = 0.1, random_state = 42). A neighbourhood graph and UMAP were computed on the resulting Harmony-corrected embedding (X_pca_harmony, first 30 components).

#### 1.3 Myonucleus classification into Fast IIA, IIX and IIB fiber types

Type II myofiber sub-identities were resolved from myosin heavy-chain expression on the log-normalized matrix. Nuclei annotated as Fast IIX, Fast IIB or MTJ were considered for reassignment, a nucleus was callable when the maximum expression across Myh2, Myh1, Myh4 and Myh7 exceeded 0.5. The dominant myosin defined the fiber type (Myh2 for IIA, Myh1 for IIX, Myh4 for IIB). Fast IIX/IIB nuclei were relabelled to their dominant fiber type, whereas junctional (MTJ) nuclei retained their identity with a subtype suffix (MTJ-IIA/IIB/IIX). Neuromuscular-junction nuclei were inherited from the scANVI annotation. Reassigned categories with fewer than 50 nuclei were dropped, and subtype assignment was validated against canonical markers (pan-fast Tnnt3/Tnnc2; Fast IIA Atp2a1/Lpl; MTJ Col22a1/Ankrd1/Csrp3). For downstream Type II analyses, the compartment was subset to Fast IIB/IIX/IIA on the integrated embedding, and residual nuclei overlapping the macrophage cluster were removed by a UMAP-coordinate cutoff.

#### 1.4 Transcriptional heterogeneity analysis

Per-nucleus transcriptional heterogeneity was quantified per cell type using an invariant-gene approach[76]. For each cell type, control (WT) and knockout (KO) nuclei were subsampled to a common size (min 500), cell types with fewer than 10 nuclei per group were skipped. An "invariant" (low-noise) gene set was defined per cell type by partitioning genes into 10 mean-expression bins and retaining, within each bin, the 10% with the lowest coefficient of variation (CV = σ/μ), controlling for the mean–variance dependence. Restricting to these low-CV genes, each nucleus’s heterogeneity score was the Euclidean distance to its own group’s mean expression profile. Group differences were assessed with a two-sided Mann–Whitney U test, corrected across cell types by the Benjamini–Hochberg FDR (*p*adj < 0.05), and effect sizes reported as Cliff ’s δ (positive δ = higher heterogeneity in KO).

### Differential gene expression and functional enrichment analysis

#### 2.1 Differential gene expression and GSEA enrichment of DEGs

Differentially expressed genes (DEGs) were called in Seurat with FindMarkers (Wilcoxon rank-sum test). Within each integrated cohort (male, female) and each cell type, cells were contrasted against WT (ident.1 = KO, ident.2 = WT) on the log-normalised expression values. p-values were adjusted for multiple testing by Bonferroni correction across all tested genes (Supplementary Tables 1 & 2). Genes with adjusted *p* < 0.01 and ∣log2 FC∣ > 1 were retained as significant and split by direction. Functional enrichment (GSEA) of the DEGs was performed by pre-ranked gene-set enrichment analysis with decoupler (dc.mt.gsea). Genes were ranked by the signed statistic log2 FC × – log10(*p*adj). Gene sets were the full mouse MSigDB collection (v2024.1.Mm symbols; GO BP/MF/CC, REACTOME, KEGG, HALLMARK, WikiPathways, BIOCARTA, PID), restricted to sets of 15–500 genes. Enrichment scores and *p*-values were computed per stratum, deduplicated to the most significant simplified term, and the common enriched pathways across strata were retained. Direction was assigned by the sign of the enrichment score and significance visualised as – log10(*p*).

#### 2.2 LLM classification of enriched pathways into functional categories

Enriched pathways shared across the sex × fiber-type strata were assigned to top-level functional categories with a large language model (Claude Haiku 4.5, model identifier claude-haiku-4-5-20251001) accessed through the Anthropic API. Raw MSigDB pathway names were submitted in batches of 60 with a system prompt describing a KEGG-BRITE-inspired taxonomy (max_tokens=4096). The model returned a JSON mapping of each pathway name to exactly one category, and unmapped names defaulted to “Other”. The seven categories and their scope are given in (Supplementary Table 3). The resulting pathway-to-category assignments were rendered as Sankey diagrams (flow width = number of pathways) and cached for reproducibility.

#### 2.3 Gene-set scoring analysis

Gene-set activity was scored per nucleus with AUCell as implemented in decoupler (dc.mt.aucell, raw=False) on the log-normalised expression; scores were written to .obs for downstream comparison. Gene sets were converted to the decoupler [77] format with species-appropriate symbol casing. Scored programmes included metabolic pathways and additional carbohydrate/glucose metabolism terms, the GOBP DNA damage-response and DNA-repair programmes, senescence signatures, and a curated atrophy gene set (PMID:31325479, 44 genes). Group differences (control vs SkM-KO) were assessed with two-sided Mann–Whitney U tests (significance thresholds * *p* < 0.05, ** *p* < 0.01, *** *p* < 0.001) and quantified with Cliff ’s δ, computed from the rank-sum statistic as δ=2𝒰∕(nxny)−1, with the “higher” group reported.

#### 2.4 Driver genes of the cellular activity scores

For each scored programme, the genes driving the AUCell score were identified within each group by the Spearman correlation between each pathway member’s expression and the per-nucleus AUCell score. Genes were ranked by absolute correlation, and the top-ranked drivers (default top 20) were visualised as per-group heatmaps of either the Spearman correlation or the mean *z*-scored expression, with gene ordering set by the strength of the score–expression association[78].

### Metacell pipeline

To construct a de-noised expression matrix for interpretable machine learning modelling, metacells were computed with SEACells (via the metashells/SeaShells wrapper)[79]. The Type II compartment (Fast IIB and Fast IIX) was subset by sex, and SEACells were built on the Harmony corrected principal components (50 components) at a target of 75 cells per metacell, computed per sample and then concatenated. Metacell expression was defined as the mean of the log-normalised expression over member nuclei. Metacells were labelled by genotype (WT=0, KO=1). This yielded 450 metacells for the male Type II compartment and 451 for the female compartment. Prior to modelling, genes that were zero in more than 80% of metacells were removed, reducing the feature space from 3000 to 1,296 genes. The resulting metacell × gene expression matrix (e.g. male: 450 × 1,296; KO=256/WT=194) and the binary genotype vector were exported (rows sorted by genotype) as the interpretable ML model inputs.

### Interpretable machine learning modeling

Latent gene modules discriminating genotype were learned with SLIDE (Significant Latent factor Interaction Discovery and Exploration)[45]. The de-noised metacell expression matrix (X) and the binary genotype label (Y: control=0, SkM-KO=1) were inputs to SLIDE, which first performs Essential Regression to decompose X into latent factors and then identifies factors (standalone/marginal and interaction terms) significantly associated with Y. Model hyperparameters (Supplementary Table 4) were selected over a δ×λ grid at a fixed sparsity (spec=0.1 for female model and 0.2 for male model), models were fit per sex.

#### 4.1 Cross-validation and AUC

Predictive performance was assessed with SLIDE::SLIDEcv using 2,000 replicates and 20-fold cross-validation, evaluated by AUC. Within each replicate the AUC of the SLIDE model (“Actual”) was compared against a permuted-label control (“Permutation”), confirming above-chance discrimination.

#### 4.2 Per-factor driver genes

For each significant factor, member genes were filtered to those with a SLIDE A-loading ≥ 0.02 and a discriminative AUC (per-gene AUC ≥ threshold or ≤ 1-threshold; threshold 0.5–0.6 depending on the factor), colour-coded by direction of association (up in SkM-KO vs up in control). Top (max A-loading) 2-3 genes in the latent factor were used to functionally annotate the latent factor using a LLM (Claude Opus 4.6)

#### 4.3 Correlation networks

Gene–gene correlation networks were built per factor from the pairwise Spearman correlations of member-gene expression across the metacells, retaining edges with ∣*r*∣ ≥ 0.25 (0.15 for Z15). Nodes were coloured by each gene’s association with genotype (per-gene ROC AUC > vs < 0.5), edges by the sign of the correlation, and node size/hubs scaled by the A-loading. Graph layouts were computed with a spring (Fruchterman–Reingold) algorithm (1,000 iterations, fixed seed).

### Human skeletal-muscle snRNA analysis

#### 5.1 Preprocessing single-nuclei SKM atlas

Cross-species analyses used a published multimodal (snRNA + snATAC) atlas of ageing human skeletal muscle (Lai et al., Nature 2024). The female Type II myofiber compartment was used (3,989 nuclei × 48,355 genes), comprising a 34-year-old “young” donor (YM2) and two 80-year-old “old” donors (OM6, OM9), with Type II subpopulations (Type II, ENOX1+, DCLK1+, SAA2+, TNNT2+) and per-nucleus pseudotime taken from the published annotation. Quality control, normalisation, feature selection, clustering and cell-type/pseudotime annotation were performed as described for the original atlas. The object was loaded pre-processed, with a log-normalised layer retained for scoring and transfer. For the SLIDE cross-prediction export, genes zero in more than 90% of nuclei were removed.

#### 5.2 Scoring, driver-gene and gene-set analysis

Human gene-set activity was scored with AUCell (decoupler, raw=False) on the log-normalised expression. Mouse gene sets were mapped to human via a stored 1:1 ortholog table (Supplementary Table 5), genes lacking a 1:1 human ortholog were dropped and per-pathway coverage recorded. Scored programmes included the mouse-derived metabolic pathway set (bridged to human symbols), and human MSigDB (v2024.1.Hs) DNA-damage/repair programmes (GOBP_DNA_REPAIR, *n* = 615; GOBP_DNA_DAMAGE_RESPONSE, *n* = 906; REACTOME_DNA_REPAIR, *n* = 332; HALLMARK_DNA_REPAIR, *n* = 150). Age groups (34 vs 80) were compared with two-sided Mann– Whitney U tests (stars as above) and Cliff ’s δ; driver genes were identified by the Spearman correlation between gene expression and AUCell score within each age group. The association of pseudotime with chronological age was assessed by linear regression (R2, *p*). PDK4 expression was summarised as mean ± 95% confidence interval (1.96×SEM).

#### 5.3 Cross-species transfer pipeline

The mouse SLIDE fuel-switch module (Z12) was projected onto the human data with a cross-species transfer of the trained mouse SLIDE model as described here. The mouse model’s A-loadings and the SLIDE model parameters were read from the female mouse SLIDE run (control vs SkM-KO). For a human expression matrix X (column *z*-scored as in SLIDE), latent-factor scores were obtained by the closed-form Essential-Regression projection, with no retraining. The projection reproduced the stored mouse *z*-matrix (Pearson *r* ≈ 1) as a sanity check. Because the A-loadings are defined in mouse gene space, each mouse gene’s column was filled with the human cell’s expression of its 1:1 ortholog (log-normalised), or zero where no orthologue existed, and orthologue coverage was reported. The transferred Z12 score (sign-oriented so that higher values correspond to older donors) was written per nucleus as the fuel-switch score and compared between age groups (Cliff ’s δ; violin plots). For cross-prediction, a linear-regression classifier was fit on the mouse metacell latent factors (significant factors vs genotype) and applied unchanged to the human latent factors (label: 80=1, 34=0). The mouse self-AUC and the mouse to human transfer AUC were computed with roc_auc_score (transfer AUC ≈ 0.629). The leading Z12 driver genes (ordered by A-loading) were displayed as a heatmap of mean *z*-scored expression at age 34 versus 80, with an A-loading side bar coloured by the mouse direction of association.

### *In-silico* perturbation of PDK4 in humans

The effect of perturbing candidate driver genes on the human ageing trajectory was evaluated *in-silico* with MaxToki[80], a decoder-only transformer model of cell-state ageing trajectories. Context “paragraphs” (~8,000 tokens) were assembled from young (34, YM2) Type II nuclei sampled along pseudotime. For each aged (80, OM6+OM9) query cell, the rank of a gene of interest was perturbed by either inhibiting or over-expressing, and the resulting change in predicted pseudotime scored as $\Delta t=t_{\text{perturbed}}-t_{\text{baseline}}$ (negative Δt=rejuvenating). PDK4, IRS2 and NR4A3 were each tested under inhibition and over-expression (*n* = 2,000 query cells per condition), pooling all 80-year-old nuclei.

### AKG Diet Administration

Calcium α-ketoglutarate-supplemented diet (2%) was purchased from Dyets Inc. (DYET# 611805). Mice were started on the α-ketoglutarate-supplemented diet at 6 months of age and maintained on the diet for 3 months. At 9 months of age, behavioral and functional assessments performed and were subsequently sacrificed for further analysis.

### Open field test

Versamax Open Field Arenas (40 cm by 40 cm by 40 cm; Omnitech Electronics, OH, United States) were housed in sound-attenuated chambers with lighting consistent with the housing room. Following acclimation to the testing room as described above, mice were placed individually into the center of the arena, and infrared beams recorded distance traveled (centimeter)/center time. Data were collected in 5 min intervals for 60 min.

### Rotarod

An accelerating rotarod (Ugo-Basile, model 47600) was used for this test. Lighting in the testing room was consistent with the housing room. Following acclimation to the testing room as described above, the mice were placed on the rotating rod (4 rpm), which accelerated up to 40 rpm over 300 s. Each mouse was subjected to three consecutive trials with an approximate 1-min intertrial interval to clean the rod between trials. Latency to fall (seconds) was measured. Subjects that fell upon initial placement on the rod before acceleration started were scored as 0 s for that trial.

### Grip strength

For grip strength, mice were weighed and acclimated for at least 1 hour before the test. Grip strength was assessed using the Bioseb grip strength meter (model # BIO-GS3 Bioseb Inc., Vitrolles, France) equipped with a grid suited for mice (100 mm by 80 mm, angled 20°). For forepaw and all paws grip strength testing, the mice were lowered toward the grid by their tails to allow for visual placing and for the mice to grip the grid with their paws. The subjects were firmly pulled horizontally away from the grid (parallel to the floor) for six consecutive trials with a brief (<30 s) rest period on the bench between trials. Trials 1 to 3 tested only the forepaw grip, whereas trials 4 to 6 included all four paws. The average of the three forepaw trials and the average of the three four-paw trials were analyzed with normalization for body weight.

### Metabolic cage studies

The major determinants of energy balance were measured in the Sable Systems Promethion Multi-plexed Metabolic Cage system, including feeding, activity, EE by indirect calorimetry, and respiratory exchange ratio. The mice were individually housed during metabolic cage studies. Studies consisted of 72 hours of cage time where the first 24 hours were considered acclimation, and the subsequent 48 hours were used to calculate the 24-hour and 12-hour light and dark cycle averages and totals for each parameter measured.

### In vivo muscle contractile testing

The surgically exposed peroneal nerve was stimulated using an electrode to induce tibialis anterior (TA) dorsiflexion, which was recorded via a force transducer–equipped footplate. Three contractile testing paradigms were performed. First a single-twitch protocol consisting of six single-pulse stimulations delivered at 5-second intervals. Second, a force–frequency protocol was used in which nine tetanic stimulations (350 ms duration) were applied at increasing frequencies (10, 30, 50, 80, 100, 120, 150, 180, and 200 Hz) with 2-minute rest intervals between stimuli. Third, a fatigue–recovery protocol consisting of 104 tetanic stimulations (100 Hz, 350 ms) delivered every 4 seconds to induce muscle fatigue followed by recovery assessments at 5 and 10 minutes after completion of the fatigue protocol using the same stimulation parameters.

### Muscle cryopreservation, cryosectioning and IF Staining

Freshly dissected muscles were mounted on corks with OCT gel and frozen by immersing in liquid nitrogen-cooled 2-methylbutane for 60 seconds. Cryosectioning was performed on a Cryostar NX50 HOPD. Serial muscle sections (10μm thickness) were collected on microscope slides, with a representative set of cross-sections on each slide. Slides were fixed in 2% paraformaldehyde (PFA) for 10 minutes at room temperature, then washed three times with 1x phosphate-buffered saline (PBS) for 2 minutes each. Next, they were permeabilized with 0.1% Triton X-100 in PBS for 15 minutes at room temperature, followed by blocking in 0.1% Triton X-100 supplemented with 3% bovine serum albumin (BSA) in PBS for 1 hour at room temperature. Primary antibodies (Table X) were diluted in 0.1% Triton X-100, 3% BSA, and 5% normal goat serum (NGS) in PBS and incubated on the slides overnight at 4 °C. Following three washes with PBS (2 minutes each), cells were incubated with secondary antibodies (Table X) diluted in the same buffer for 1 hour at room temperature in the dark. Finally, slides were washed three times with PBS and coverslips were mounted using fluoromount with DAPI (catalog no#: 00-4959-52, Invitrogen) before imaging.

### Fiber Type staining, imaging, and analysis

Frozen muscle sections were first allowed to air dry completely at room temperature. Slides were then incubated for 1 hour at room temperature in a blocking solution containing 0.5% Triton X-100, 1% BSA, and 10% goat serum in PBS. Primary antibodies against laminin, type I, type IIA fibers, and type IIB fibers were prepared in a solution of 0.1% Tween-20, 1% BSA, and 5% goat serum in PBS, according to the dilutions listed in Table X. This antibody mixture was applied to the sections and incubated overnight at room temperature. Following incubation, slides were washed three times in PBS for 5 minutes each. Sections were then fixed in 10% neutral buffered formalin for 10 minutes and washed again three times in PBS. Appropriate secondary antibodies (goat anti-mouse IgG2b, IgM and IgG1, and goat antirabbit; Invitrogen) were prepared at a 1:500 dilution in the same buffer used for primary antibodies. Sections were incubated with secondary antibodies for 1 hour at room temperature, followed by three additional PBS washes. Finally, slides were washed, air dried, and coverslipped using Flouromount-G (Catalog #: 0100-01) mounting medium. Imaging for fiber type was performed on a Zeiss Axio Observer microscope with a motorized stage at 20x magnification. Tiled images were taken of the entire muscle sections, then the Zen Blue software was used to stitch the images together. One section was captured for each animal. Analysis was conducted using MuscleJ, an automated ImageJ plugin (SOURCE).

### Trichrome staining and imaging

Tissue sections were fixed in acetone for 10 minutes and washed twice in phosphate-buffered saline (PBS) for 3 minutes each. Sections were then briefly immersed in 95% ethanol and air-dried for 30 minutes. Subsequently, samples were incubated in Bouin’s solution at 56 °C for a minimum of 30 minutes, followed by rinsing in running tap water until clear (~5 minutes) and a final rinse in deionized water. Sections were stained with Weigert’s hematoxylin for 3 minutes and washed in tap water for 2 minutes, followed by staining with Biebrich scarlet/acid fuchsin for 8 minutes and rinsing in deionized water. Sections were then treated with phosphomolybdic/phosphotungstic acid for 10 minutes and directly transferred, without rinsing, to aniline blue for 8 minutes. Sections were subsequently rinsed in deionized water and treated with acetic acid for 5 minutes. Finally, sections were rapidly dehydrated through graded ethanol solutions (95% ×2, 100% ×2), cleared in xylene for 1 minute, and mounted using Clearvue mounting medium.

Imaging was performed using a Keyence BZ-X810 at 10x magnification and MoticEasyScan at 40x magnification. Images were analyzed with ImageJ by using color deconvolution to isolate the collagen signal and quantifying percent area per field.

### Cell culture

C2C12 cells were obtained from ATCC (cat# - CRL1772) and were cultured in Dulbecco’s modified Eagle medium (DMEM) (Gibco-11965-084) with 10% FBS (ThermoFisher, cat# - A5670701), 1% penicillin–streptomycin (ThermoFisher, cat# −15140122). C2C12 differentiation was initiated by switching to high-glucose DMEM containing 2% horse serum (Gibco, cat# −26050088) when they were 80-90% confluent.

### Cell treatments

After 48 h of differentiation, cells were treated with 0.5 μM doxorubicin (Sigma, cat# -D5220) for 2 h, followed by replacement with fresh culture medium. For PDK4 inhibition and α-ketoglutarate (AKG) supplementation experiments, cells were treated with 0.5 μM doxorubicin for 2 h in the presence of either 1 μM PDK4 inhibitor or 5 mM AKG. After 2 h, doxorubicin was removed by medium replacement, whereas PDK4 inhibitor or AKG treatment was maintained for the remainder of the experiment. All downstream analyses were performed 96 h after the initial treatment.

### In vitro staining, imaging, and analysis

Cells were fixed in 2% paraformaldehyde (PFA) for 10 minutes and washed three times with 1× phosphate-buffered saline (PBS) for 2 minutes each. If staining was not performed immediately, fixed samples were stored in PBS at 4 °C for up to two weeks. Cells were then permeabilized with 0.1% Triton X-100 in PBS for 15 minutes at room temperature, followed by blocking in 0.1% Triton X-100 supplemented with 3% bovine serum albumin (BSA) in PBS for 1 hour at room temperature. Primary antibodies (Table X) were diluted in 0.1% Triton X-100, 3% BSA, and 5% normal goat serum (NGS) in PBS and incubated with the cells overnight at 4 °C. Following three washes with PBS (2 minutes each), cells were incubated with secondary antibodies (Table 1) diluted in the same buffer for 1 hour at room temperature in the dark. Finally, cells were washed three times with PBS, incubated with Hoechst (catalog #: 62249, Thermo Scientific) for 5 minutes, and washed twice more before imaging. Imaging was performed at 10X magnification using a Nikon Eclipse microscope. All image analyses were carried out using ImageJ.

### Quantitative PCR

For analysing gene expression by qPCR, RNA was isolated from samples using the TRIZOL/chloroform extraction method. cDNA was generated using 1ug RNA per reaction as described by the manufacturer’s protocol (Fisher Scientific, cat# - 50-997-721). qPCR was performed using Power Track SYBR Green Master Mix (Thermo-A25742). Genes of interest were normalized to β-actin. Primers are listed in Supplementary Table 2.

### Immunoblotting

Cell lysates were prepared by the addition of 4× Laemmli sample buffer and β-mercapitoethanol. Samples were then boiled at 95 °C for 10 min before being resolved by 4–20% SDS–PAGE gel electrophoresis using standard protocols. After transfer, nitrocellulose membranes were placed on a shaker in blocking buffer (5% BSA in PBS) for 1 h at room temperature. Blots were incubated with primary antibodies (Table 1) overnight at 4 °C before being washed and incubated with secondary antibodies for 1 h at room temperature (Table 1). Blots were imaged using SuperSignal^™^ West Pico PLUS Chemiluminescent Substrate (ThermoFisher, cat#-34580) on a Bio-Rad ChemiDocimaging system. Fiji (v.1.54p) (ImageJ, NIH) was used for blot analyses.

### Oleate oxidation rate assay

Cells were plated in 6-well plates and treated with vehicle, doxorubicin alone, or doxorubicin in combination with PDK4i. On the day of the experiment, growth medium was replaced with reaction medium consisting of serum-free DMEM supplemented with an oleic acid-BSA complex (Sigma-Aldrich, Cat#O3008), delivering 0.43 nmol BSA and 2.67 nmol oleate per well, and 1 μCi 1-^14^C-oleate (Revvity, Cat#NEC317250UC). After a 1-hour incubation at 37°C, the reaction medium and cells were collected for analysis. The rate of oleate oxidation was determined from the ^14^CO_2_ released into the reaction medium and the specific activity of 1-^14^C-oleate in the reaction, normalized to total protein (PMID: 24862277).

### Calcium Transient Imaging and Analysis in Engineered C2C12 Skeletal Muscle Tissues

#### Engineered skeletal muscle tissue fabrication and treatment

Engineered skeletal muscle tissues were fabricated from C2C12 myoblasts (ATCC, CRL-1772) using a previously published protocol[81]. Briefly, 3 × 10^6^ C2C12 cells were suspended in proliferation medium consisting of DMEM supplemented with 10% FBS, followed by the addition of 40% Matrigel and 1 mg/mL rat tail collagen type I. A total of 250 μL of the cell–hydrogel mixture was cast into a sterilized muscle construct frame and incubated at 37°C for 45 min to allow gelation. The muscle construct mold contained two posts on each side to facilitate uniaxial alignment of the developing myotubes. Constructs were maintained in proliferation medium for 1 day and subsequently cultured in differentiation medium for 11 days. Four experimental conditions were evaluated: vehicle control, doxorubicin (Doxo), α-ketoglutarate (α-KG), and combined Doxo plus α-KG. On day 11 of differentiation, muscle constructs were treated with 0.5 μM doxorubicin for 2 h. For α-ketoglutarate (α-KG) experiments, constructs were co-treated with 0.5 μM doxorubicin and 5 mM α-KG for 2 h, after which doxorubicin was removed, and α-KG treatment was continued. Vehicle-treated constructs underwent the corresponding vehicle treatment. A total of three tissue constructs were analyzed per experimental group. All downstream analyses were performed 48 h after the initial treatment.

#### Calcium indicator loading

Intracellular calcium was reported with the fluorescent indicator Cal-520 AM (AAT Bioquest, Cat. No. 21131; 1 mg). Cal-520 AM was reconstituted in DMSO to a concentration of 5 mM. For calcium indicator loading, 2 μL of the Cal-520 AM stock solution was added directly to the PDMS well containing approximately 250 μL of culture medium, resulting in a final indicator concentration of approximately 40 μM. Tissues were incubated with indicator for 3 hours at 37°C before imaging. Immediately prior to imaging, each tissue was transferred, while remaining within its PDMS well, to a well of a 6-well plate filled with 8 mL of fresh Tyrode's buffer (pH 7.4), pre-warmed to 37°C.

#### Field stimulation and imaging conditions

Tissues were paced by field stimulation delivered through a custom platinum wire electrode assembly positioned in the imaging well and driven by a GRASS S44 stimulator at 80 volts and 1 pulse per second, giving a nominal 1 Hz pacing rate. Additional GRASS stimulator settings: Delay – 1 ms, Duration – 40 ms, Single pulses, and Repeat mode. Sample temperature was held at 37 degrees Celsius using a heated stage; the stage controller setpoint was 42 degrees Celsius, which was empirically determined to deliver 37 degrees Celsius at the tissue. Pacing rate recovered from the recorded traces was 1.04 Hz, consistent with the 1 pulse per second command, and the coefficient of variation of the inter-transient interval was used to confirm capture on every recording.

#### Widefield fluorescence image acquisition

Calcium transients were recorded on a Leica widefield fluorescence microscope (THUNDER class) equipped with a Leica K8 camera and operated through LAS X, using a 1X objective. Cal-520 was excited at 470 nanometers and emission collected at 525 nanometers through an ET GFP filter cube, with illumination intensity set to 100 in LAS X relative units. Single-channel time series were acquired with an exposure time of 5 milliseconds, camera sensitivity mode 11-bit, gain set to sensitivity, no electron multiplication, and no frame averaging or accumulation. Images were acquired with 2 by 2 binning at 1024 by 1024 pixels, giving a pixel size of approximately 3.57 μm and a field of view of approximately 3.65 by 3.65 mm. The frame interval recorded in the image metadata was 0.010578 seconds, corresponding to 94.5 frames per second, and recordings spanned approximately 10 to 14 seconds (964 to 1285 frames), capturing 10 or more consecutive paced transients per tissue. Data were stored as 11-bit values in 16-bit unsigned integer format.

### Calcium transient analysis

#### Region definition

Image stacks were analyzed with a custom Python pipeline based on prior work[82] (Also available at www.ShiwarskiLab.com). Each recording was analyzed at three spatial levels, which were reported alongside one another rather than one being treated as definitive. First, a whole-tissue mask was obtained by Otsu thresholding the temporal mean projection, removing objects smaller than 2000 pixels and filling holes. Second, an active-tissue region was defined as the subset of that mask in which the per-pixel transient amplitude reached a fixed threshold of 0.10 in units of relative fluorescence change, computed as the difference between the systolic and diastolic frame averages divided by the diastolic average. This second region preserves whole-field spatial coverage while excluding non-firing pixels, which otherwise dilute the spatially averaged signal. Third, regions of interest were placed on a 20 by 20 grid of square cells (approximately 150 μm, per side), and 10-15 ROIs per tissue were selected automatically by ranking every qualifying cell using a weighted combination of a fine-scale focus measure, temporal standard deviation, and transient amplitude. Cells were disqualified for insufficient tissue coverage, proximity to the tissue border, absence of detectable signal, or inadequate focus. Each selected region of interest was then refined to its signal-bearing pixels by thresholding the temporal standard deviation within that region, and the surviving pixel fraction was recorded for quality control.

#### Baseline fluorescence and relative fluorescence change

Because the relative fluorescence change is defined as the difference between instantaneous and baseline fluorescence divided by baseline fluorescence, the baseline definition scales every reported amplitude and was therefore specified explicitly rather than taken as a percentile of the whole trace. Baseline fluorescence was defined as the median raw fluorescence at the diastolic transient feet during pacing, located by a two-pass procedure: a provisional relative fluorescence trace was used to detect transients and their feet, baseline was computed from the raw trace at those frames, and the relative fluorescence trace was then recomputed. This definition is independent of how many unpaced frames a recording happens to contain, which a percentile-based baseline is not. The diastolic frames were determined once per tissue, from whichever region detected the most transients, and applied to every region of that tissue, so that all regions of a tissue share a single baseline definition. The resolved baseline value, the rule used, the number of frames contributing to it, and the region supplying those frames were recorded for every region.

#### Transient detection and parameter extraction

Transients were detected by prominence-based peak detection, with the prominence threshold set to the larger of an absolute floor and a fixed fraction of the trace range, and a minimum peak separation of 0.45 seconds, which is below the 0.96 second pacing interval. The threshold actually applied to each region was recorded, together with a flag indicating whether the absolute floor or the proportional term was the binding constraint. The foot of each transient was defined as the activation onset: searching backwards from the peak within a window bounded by the preceding peak and 35 percent of the median inter-peak interval. The foot is the last frame at or below the window's diastolic level, taken as its 10th percentile, plus 5 percent of the transient amplitude. This onset definition was adopted in preference to the local minimum within the interval because once a transient has decayed the trace occupies a flat, noise-dominated diastolic plateau, and the minimum of such a segment falls at an essentially arbitrary frame within the search window, making foot-referenced measurements a function of the window length rather than of the underlying signal.

For every transient the following were measured: amplitude, as the relative fluorescence change at the peak minus that at the foot; time to peak, from foot to peak; the 10 to 90 percent rise time on the upstroke; the decay time constant from a single-exponential fit spanning peak to the subsequent foot, with the coefficient of determination reported so that poor fits could be identified; and the times from the foot to 50 and 90 percent recovery toward baseline, reported as CaTD50 and CaTD90. Pacing frequency was taken as the inverse of the mean inter-transient interval and its regularity as the coefficient of variation of that interval. Rise times falling within two frame intervals of the sampling limit were flagged as unresolved, in which case time to peak was used in preference. A peak-aligned ensemble average was constructed for each region across its beats, spanning 0.2 seconds before to 0.6 seconds after the peak; peak alignment rather than foot alignment was used for amplitude reporting because foot alignment smears the peak across the ensemble and understates its height.

#### Contractile Displacement Analysis

Tissue contraction was quantified directly from time-lapse image sequences without performing two-dimensional image registration. For each recording, the tissue was cropped to its bounding box, and each frame was converted to a gradient-magnitude image to emphasize tissue boundaries while minimizing the influence of calcium-dependent changes in fluorescence intensity. The gradient image was then projected along the two image axes by summing pixel intensities across rows and columns, generating one-dimensional spatial intensity profiles for each frame.

Each profile was z-score normalized and cross-correlated with a reference profile generated as the median of the first 20 frames of the recording. Tissue displacement along each image axis was determined from the location of the maximum correlation within a ±30-pixel search range, with sub-pixel refinement of the correlation peak. The two orthogonal displacement components were combined to obtain the Euclidean displacement magnitude after removal of their respective median offsets, generating a frame-by-frame tissue displacement trace.

To reduce high-frequency noise while preserving the temporal characteristics of contraction, the displacement trace was first median-filtered over 21 ms and subsequently smoothed using a second-order Savitzky–Golay filter with a 50-ms window. Contraction events were identified using prominence-based peak detection. The prominence threshold was defined as the greater of an absolute displacement threshold of 0.15 pixels or 25% of the displacement-trace range, with a minimum inter-peak separation of 0.45 s.

For each detected contraction, displacement amplitude was calculated as the difference between the peak displacement and the 10th percentile of the displacement trace. The mean amplitude of all detected contractions within a recording was used as the tissue-level contractile amplitude. Contraction frequency was calculated as the inverse of the mean interval between consecutive contraction peaks, whereas contraction regularity was quantified using the coefficient of variation of the inter-contraction intervals.

To account for spatial heterogeneity in tissue contraction, the same displacement analysis was independently applied to four spatially defined tissue blocks arranged as a 2 × 2 grid. The minimum and maximum displacement amplitudes among the four blocks were retained to characterize the spatial range of contractile activity across the tissue.

When calcium peak frames were available, the temporal delay between each calcium peak and the subsequent displacement peak was calculated as a measure of electromechanical coupling. Displacement values were converted from pixels to micrometers when the image pixel size was available from the acquisition metadata; otherwise, displacement was reported in pixels.

### Repair Assisted Damage Detection assay (RADD ASSAY)

RADD ASSAY is performed on TA muscle sections as described previously.[83]

### Oxygen Consumption Rate (OCR) Measurements

OCR was assessed using the Resipher real-time metabolic analysis platform (Lucid Scientific) as previously described[84]. Approximately 10,000 C2C12 mouse skeletal muscle cells were seeded in 96-well plates and monitored continuously for 24 h under standard culture conditions (37 °C, 5% CO_2_). Cells were subsequently differentiated into myotubes over 48hrs using our standard differentiation protocol. Following differentiation, cells were assigned to the indicated treatment groups: control, doxorubicin (DOXO), PDK4 inhibitor (PDK4i), or DOXO + PDK4i (n = 5–6 wells per group). OCR was continuously monitored throughout the treatment period to assess changes in cellular respiration. At the end of the experiment, total protein content was quantified for each well and used to normalize OCR measurements. All bioenergetic comparisons were performed using protein-normalized OCR values.

### Statistical analysis of experimental data

Statistical analyses were performed using GraphPad Prism (v11.0.0). For normally distributed data, comparisons were made using Student’s t-test or one-way ANOVA followed by Tukey’s post hoc multiple comparisons test. For non-normally distributed data, the Mann–Whitney U test or Kruskal–Wallis test with Dunn’s post hoc multiple comparisons was used. For testing significance between sexes and genotypes, a 2-way ANOVA test or a *p*-value less than .05 was considered statistically significant. Data are presented as mean ± standard error of the mean (SEM).

## Supplementary Material

This is a list of supplementary files associated with this preprint. Click to download.

- supplementaryfigures.pdf

**Supplementary Figure 1. ERCC1 deletion strategy and age-dependent DNA lesion accumulation in naturally aged skeletal muscle.** (A) Schematic of the conditional ERCC1 knockout allele generated by CRISPR-Cas9 genome editing, with exon 5 flanked by loxP sites. Cre-mediated deletion of exon 5 generates a frameshift and premature stop codon, producing the SkM-KO allele. (B) Representative BaseScope in-situ hybridization(ISH) images in TA muscle sections from male control and SkM-KO mice. Quantification of ERCC1 signal (dots/25μm) is shown for both female and male control and SkM-KO mice. Nuclei are counterstained with DAPI. Scale bars= 25 μm. (C) Representative RADD images and quantification of the OxRADD, T4PDG and UDG lesions in skeletal muscle sections from female and male naturally aged mice at 4, 23 and 28. Line graphs show fluorescent intensity (arbitrary units) as a function of age for each lesion class and sex. Nuclei are counterstained with DAPI. Fluorescence intensity was quantified and expressed in arbitrary units. Scale bars=100 μm. *p<0.05, ***p<0.001, ****p<0.0001. Sample size reflected in bar plots. *t-*test for two-group comparison. One-way ANOVA for multiple group comparison.

**Supplementary Figure 2.** Muscle morphology in the tibialis anterior, quadriceps, and soleus of female and male SkM-KO mice. (A) Quantification of type IIA fibers and collagen area in the tibialis anterior (TA) muscle of female and male control and SkM-KO mice. (B) Quantification of mean fiber area (mm^2^), type IIA and IIB fiber percentage in the quadriceps of female and male control and SkM-KO mice. (C) Mean fiber area (mm^2^) in the soleus female and male control and SkM-KO mice, serving as a type I fiber specificity control confirming that *Myl1-Cre*-mediated ERCC1 deletion does not affect slow-twitch fiber populations. *p<0.05, ***p<0.001, ****p<0.0001. Sample size reflected in bar plots. *t*-test for two-group comparison. One-way ANOVA for multiple group comparison.

**Supplementary Figure 3. Young female SkM-KO mice show preserved muscle structure and gross contractile function with selective early impairment of stimulation frequency response. (A)** Body weight (g), tibialis anterior (TA) bulk muscle cross-sectional area (CSA, mm^2^), normalized tetanic force (mN/mm^2^), stimulation frequency response (Hz), time to maximum contraction following single-twitch stimulation (s), half-relaxation time (s) , and percent initial response following a fatigue protocol, assessed in young (4-5 mo) female control and SkM-KO mice. All parameters are preserved except stimulation frequency response, which is significantly reduced in young SkM-KO females (p<0.05), identifying Ca^2+^ handling impairment as the earliest detectable functional consequence of ERCC1 loss, preceding atrophy and gross force decline. *p<0.05, ***p<0.001, ****p<0.0001. Sample size reflected in bar plots. *t*-test for two-group comparison. One-way ANOVA for multiple group comparison.

**Supplementary Figure 4. Marker-gene based cell-type annotation, senescence/DNA-repair scoring and metabolic-pathway rewiring of Type II myofibers.** (A) UMAP embeddings of the single-nuclei atlas coloured by expression of representative Type II fiber markers (Myh4, Fast IIB; Myh1, FastIIX; Myh2, Fast IIA), alongside a dot plot of canonical marker genes across the 13 annotated cell populations. Dot size denotes the fraction of nuclei expressing each gene and colour intensity the mean expression. (B) Senescence activity scores assessed using two independent gene signatures (Senmayo and Sensig) in Fast IIB myonuclei from female and male control and SkM-KO mice. The absence of large senescence score increases in SkM-KO mice indicates that ERCC1 loss in postmitotic myofibers does not engage canonical senescence programs at this timepoint. (C) Metabolic pathways upregulated in female Fast IIB nuclei of SkM-KO mice: xenobiotic(Cliff’s*δ*=0.342), pyruvate (*δ*=0.241), glycogen (*δ*=0.211), carbohydrate (*δ*=0.190), phospho-lipid (*δ*=0.124) and fatty-acid (*δ*=0.039) metabolism are all higher in SkM-KO. (D) Metabolic pathways downregulated in female Fast IIB nuclei of SkM-KO mice: glycosaminoglycan (*δ*=−0.028) and purine (*δ*=−0.007) metabolism are higher in control. (E) Per-group Spearman-correlation heatmaps of the leading driver genes of the pyruvate (Me3, Pdk4, Pcx, Ldhb), carbohydrate (Ugp2,Gbe1, Hk2, Gys1 and others) and glycogen (Ugp2, Gbe1, Gys1, Phka1, Ppp1r3c) metabolic scores in control versus SkM-KO female Fast IIB nuclei. Activity scores were computed with AUCell and group differences assessed by Mann–Whitney U test with the Cliff’s *δ* effect size. *p < 0.05, ***p < 0.001.

**Supplementary Figure 5. SLIDE model cross-validation and male latent-factor networks.** (A) Predictive performance (AUC) of the SLIDE latent-factor model versus random label permutation across 25 cross-validation data splits, shown separately for female (median = ~0.92) and male (~0.80) metacells. The permutation control performs at chance (AUC=0.5) far exceeding the chance-level performance of random label permutation (AUC ≈ 0.52 for both sexes), confirming that the identified latent factors capture true biological signal. (B) Gene-correlation networks of male SkM-KO standalone modules: Z15 (Regulation of Energy Metabolism and Muscle Adaptation), in which Pdk4 and Ucp3 show higher expression in SkM-KO (lighter nodes) while Egr1, Abra, and Nav2 show higher expression in control, indicating that PDK4 occupies a qualitatively different network context in males compared to the FAO fuel switch module identified in females; and Z29 (Calcium Signalling), centred on Il15 and Slc8a1. Dark nodes indicate higher expression in control (WT); light nodes indicate higher expression in SkM-KO; grey nodes indicate non-differential expression.

**Supplementary figure 6. PDK4 inhibition preserves myotube formation following DNA-damage.** (A) Immunoblot analysis of PDH, phosphorylated PDH (Ser 293) in TA lysates from female control and SkM-KO mice, confirming PDK4-mediated PDH phosphorylation *in vivo*. (B-C) Fractional enrichment of pyruvate, lactate (B), CoASH, CoASSG (C) in TA muscle from male control and SkM-KO mice by LC-MS, showing increased acetyl-CoA (p<0.05) consistent with preserved PDC activity and enhanced pyruvate flux in males. (D) Immunoblot analysis of γH2AX expression in C2C12 myotubes following doxorubicin treatment for the indicated time points. (E) Representative immunofluorescence images and quantification of C2C12 myotubes stained for PDK4 following doxorubicin and/or PDK4 inhibitor treatment. Nuclei were counterstained with DAPI. Scale bars, 25 μm. (F) Lactate levels measured in C2C12 myotubes following doxorubicin treatment in the absence or presence of PDK4 inhibitor. (G) Representative immunofluorescence images of C2C12 myotubes stained for myosin heavy chain (MHC) following cisplatin and 5 fluorouracil and/or PDK4 inhibitor treatment. Nuclei were counterstained with DAPI. Quantification shows the percentage of multinucleated MHC-positive myotubes. Scale bars, 100 μm. *p<0.05, ***p<0.001, ****p<0.0001. Sample size reflected in bar plots. T-test for two-group comparison. One-way ANOVA for multiple group comparison.

**Supplementary Figure 7. α-Ketoglutarate supplementation improves molecular and functional phenotypes associated with skeletal muscle aging.** (A) High-resolution respirometry in isolated TA myofibers from male control and SkM-KO mice. Oxygen flux (pmol O_2_·s^−1^·mg quad^−1^) was measured under pyruvate + malate (PM) and pyruvate + malate + glutamate (PMG) substrate conditions. No significant differences are observed in males under either substrate condition, consistent with preserved pyruvate oxidation and absence of glutamate anaplerotic dependency in male SkM-KO muscle. (B) qPCR quantification of p21 and Atrogin-1 mRNA expression (fold change) in TA muscle from female control and SkM-KO mice with or without αKG dietary supplementation. αKG supplementation significantly reduces Atrogin-1 expression in SkM-KO mice (p<0.0001) without significantly altering p21 levels, indicating that αKG attenuates the atrophy program without affecting the DNA damage checkpoint/senescence. (C) Quantification of multinucleated eMHC-positive fiber percentage in C2C12 myotubes across six treatment conditions testing αKG alone, doxorubicin + αKG, αKG + PDK4 inhibitor, and doxorubicin + αKG + PDK4 inhibitor combinations. The combination of αKG and PDK4 inhibitor rescues myotube formation to the same extent as either treatment alone, indicating convergence on a shared TCA intermediate bottleneck rather than synergistic action through independent pathways (D) Representative calcium transient traces from C2C12-based three-dimensional bioengineered skeletal muscle constructs under control, doxorubicin (Doxo), and doxorubicin + αKG (Doxo-AKG) conditions. Calcium responses are expressed as ΔF/F_0_. Doxorubicin reduces transient amplitude and αKG partially restores it, consistent with quantitative analyses in main Figure 6M.

**Supplementary Figure 8. PDK4 marks an aged transcriptional pseudotime trajectory in human female Type II myofibers, with in-silico PDK4-i displaying rejuvenation.** (A) snRNA-seq-based pseudotime trajectory of human female type II myonuclei (UMAP coloured by pseudotime score, 0–100). Violin plots show pseudotime distribution stratified by chronological age (34 years: YM2; 80 years: OM6+OM9), confirming that pseudotime correlates significantly with chronological age (R^2^=0.102, p=4.75×10^−89^) (B) DNA damage response activity shows a non-monotonic trajectory with an initial plateau followed by progressive increase at higher pseudotime values, while PDK4 expression rises continuously, suggesting that PDK4 induction precedes full DDR activation along the aging trajectory. (C) Activity scoring of female Type II single-nuclei:REACTOME glycogen metabolism (*δ*=−0.526, higher at 34) declining with age. (D) Schematic of the MaxToki zero-shot perturbation pipeline. (i) Context sampling: three sampling strategies capture cells from the young (34-year-old) pseudotime trajectory to represent the young transcriptional reference state. (ii) Context paragraphs: approximately 8,000 context-cell paragraphs are built using gene rank sequences from young cells as context and 80-year-old cells as query cells. (iii) Gene perturbation: the gene of interest (e.g., PDK4) is moved to the top rank (inhibition) or bottom rank (overexpression) within the query cell's gene rank sequence. (iv) Perturbation scoring: the resulting shift in pseudotime (Δt = perturbed – baseline pseudotime) is computed across all query cells; negative Δt indicates rejuvenation toward a younger transcriptional state..

## Figures and Tables

**Figure 1.**
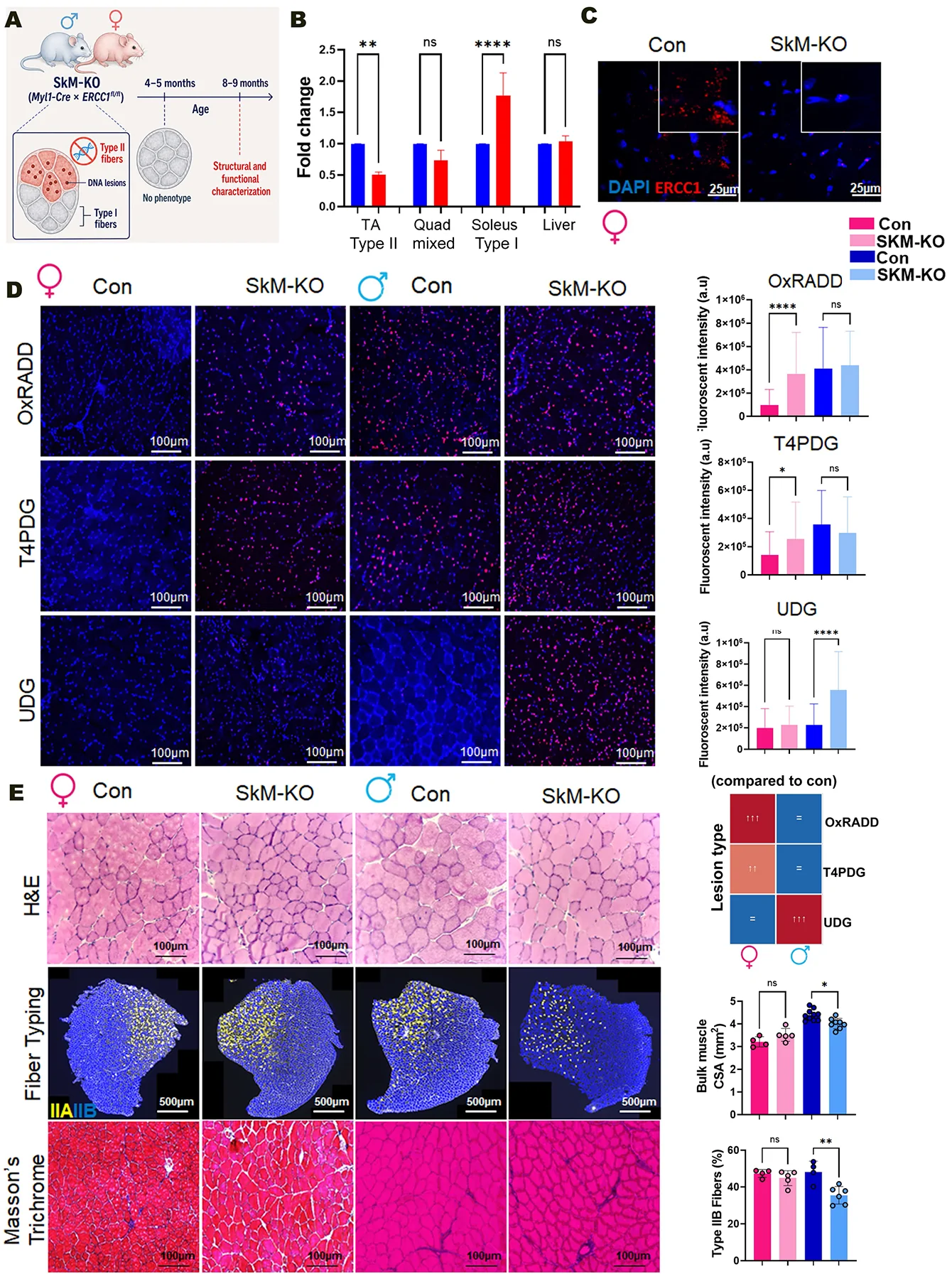
Type II fiber-specific ERCC1 deletion leads to sex-specific accumulation of DNA damage. (A) Schematic of the skeletal muscle-specific ERCC1 knockout strategy. ERCC1-floxed mice (ERCC1^fl/fl^)were crossed with Myl1-Cre mice to generate SkM-KO animals, restricting ERCC1 deletion to fast-twitch type II myofibers. The timeline indicates the period of phenotypic assessment: 4–5 months (pre-phenotypic) and 8–9 months (full structural and functional characterization). (B-C) qPCR quantification of *Ercc1* mRNA in TA (type II), quadriceps (mixed), soleus (type I), and liver, confirming muscle type II-selective deletion (B) and Basescope *in-situ* hybridization (ISH) (C). Nuclei are counterstained with DAPI. Scale bars = 25 μm. (D) Representative images and quantification of the RADD assay in skeletal muscle sections from female and male control and SkM-KO mice. OxRADD, T4PDG and UDG signals indicate oxidative DNA damage, cyclobutane pyrimidine dimer-related lesions and uracil-associated DNA lesions, respectively. Nuclei are counterstained with DAPI. Fluorescence intensity was quantified and expressed in arbitrary units. Scale bars = 100 μm. A summary matrix indicates the direction of change in each lesion type in SkM KO relative to control for each sex (↑ = increased, = unchanged) (E) Representative images of TA muscle sections from control and SkM-KO mice stained with hematoxylin and eosin (H&E), immunofluorescence fiber typing (type IIA in yellow, type IIB in blue), and Masson’s trichrome. Quantification of TA bulk muscle cross-sectional area (CSA) and the distribution of type IIA and type IIB muscle fibers is shown. Scale bars= 100 μm for H&E; Masson’s trichrome staining and 500 μm for fiber typing. *p<0.05, ***p<0.001, ****p<0.0001. Sample size reflected in bar plots. *t*-test for two-group comparison. One-way ANOVA for multiple group comparison.

**Figure 2.**
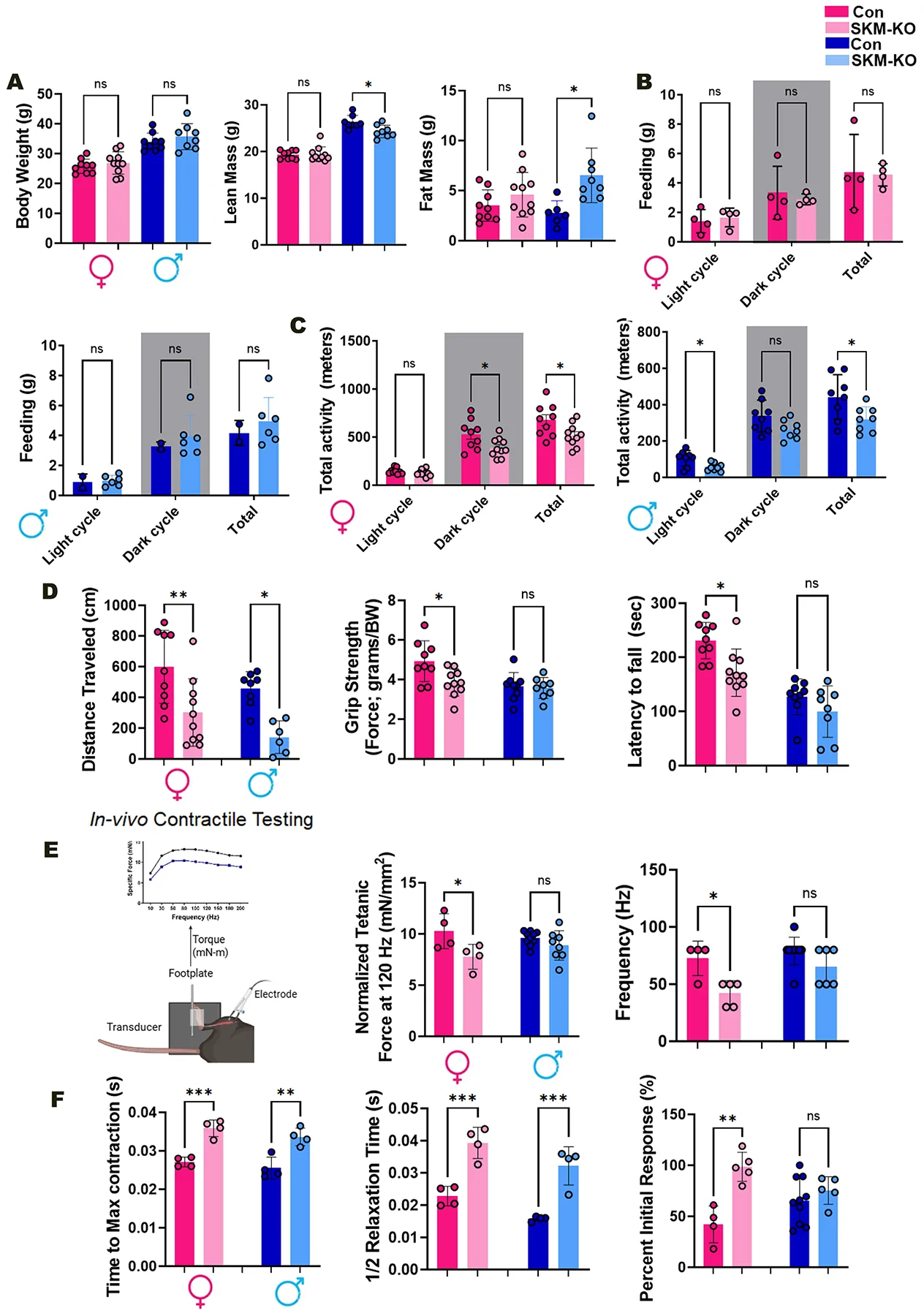
DNA damage impairs muscle function and contractility in a sex-specific manner (A) Body weight (g) and body composition [lean mass (g), and fat mass (g)] assessment, between male and female control and SkM-KO mice. (B) Food intake (g) measured during light cycle, dark cycle, and total in female and male mice housed in metabolic cages. (C) Total locomotor activity (meters) measured during light cycle, dark cycle, and total in female and male mice (D) Distance traveled (cm), forelimb grip strength (force normalized to body weight, grams/BW), and rotarod latency to fall (sec) in female and male control and SkM-KO mice. (E) A schematic of the *in vivo* TA contractile testing apparatus is shown. Normalized Tetanic force, frequency for TA stimulation. (F) Temporal contractile properties: time to maximum contraction (s), half-relaxation time (s), and percent initial response (%) following a fatigue protocol, from single twitch stimulation of the TA muscle. *p<0.05, ***p<0.001, ****p<0.0001. Sample size reflected in bar plots. T-test for two-group comparison. One-way ANOVA for multiple group comparison.

**Figure 3.**
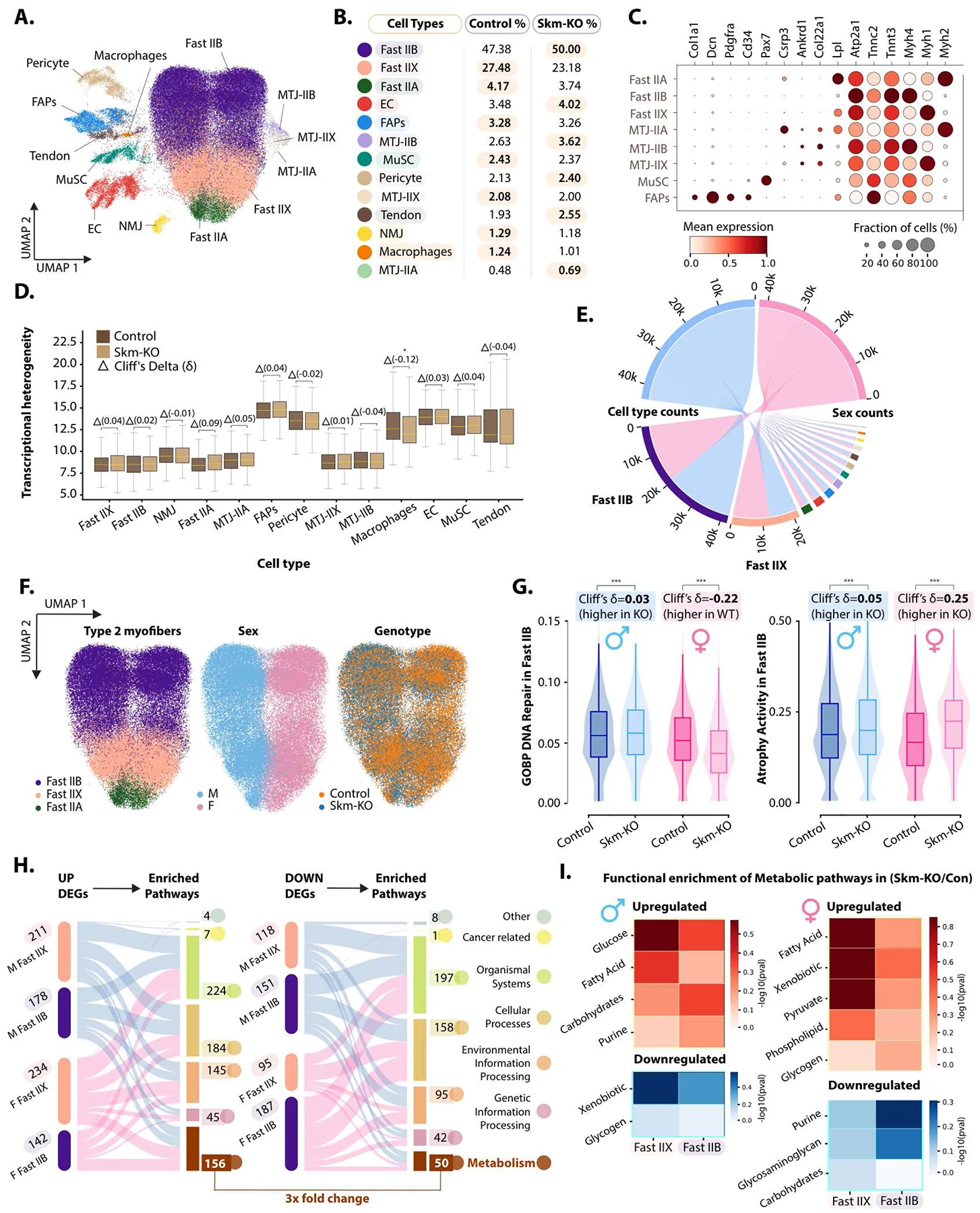
Single-nuclei RNA-seq atlas of mice skeletal muscle reveals metabolic rewiring to be a transcriptional signature of DNA-damage (A) UMAP embedding of the integrated single-nuclei RNA-seq atlas of mouse tibialis anterior (TA) muscle (male (n = 6) and female (n = 6) samples), annotated into 13 cell-type populations. (B) Relative cell-type composition in control versus SkM-KO, as a percentage of total nuclei. Type II myofibers dominate (Fast IIB 47.38% control vs 50.00% KO; Fast IIX 27.48% vs 23.18%; Fast IIA 4.17% vs 3.74%). (C) Dot plot of canonical marker genes across cell types. Dot size denotes the fraction of nuclei expressing each gene and colour intensity represents the mean expression. (D) Quantification of transcriptional heterogeneity per cell-type in control versus SkM-KO, with the Cliff’s *δ* effect size between the two conditions annotated above each pair (e.g. Macrophages *δ* = −0.12). (E) Chord diagram summarising nuclei counts by cell-type and by sex (males and females have equivalent number of cells), highlighting the predominance of Fast IIB and Fast IIX myofibers in the atlas and confirming equivalent cell numbers between male and female samples. (F) UMAP of Type II myofiber compartment (Fast IIB, IIX, IIA) coloured by cell-type, sex and genotype. (G) Pathway activity scoring in Fast IIB nuclei, split by sex. DNA repair activity score is reduced in female SkM-KO nuclei (Cliff's δ = −0.22) with a negligible effect in males (δ = 0.03), while atrophy activity is higher in SkM-KO cells (males δ = 0.05, females δ = 0.25) (H) Sankey plots mapping differentially expressed genes (DEGs between SkM-KO vs control) to GSEA enriched pathways separated by sex and fiber type. Metabolic pathways show a 3-fold difference between enrichment in upregulated DEGs vs downregulated DEGs, establishing metabolic rewiring as the dominant transcriptional hallmark of DNA damage (I) Heatmaps of metabolic-pathway enrichment in DEGs (SkM-KO vs control), shown separately for males and females across Fast IIX and Fast IIB cell-types and coloured by significance of enrichment (– log10(*p*)).

**Figure 4.**
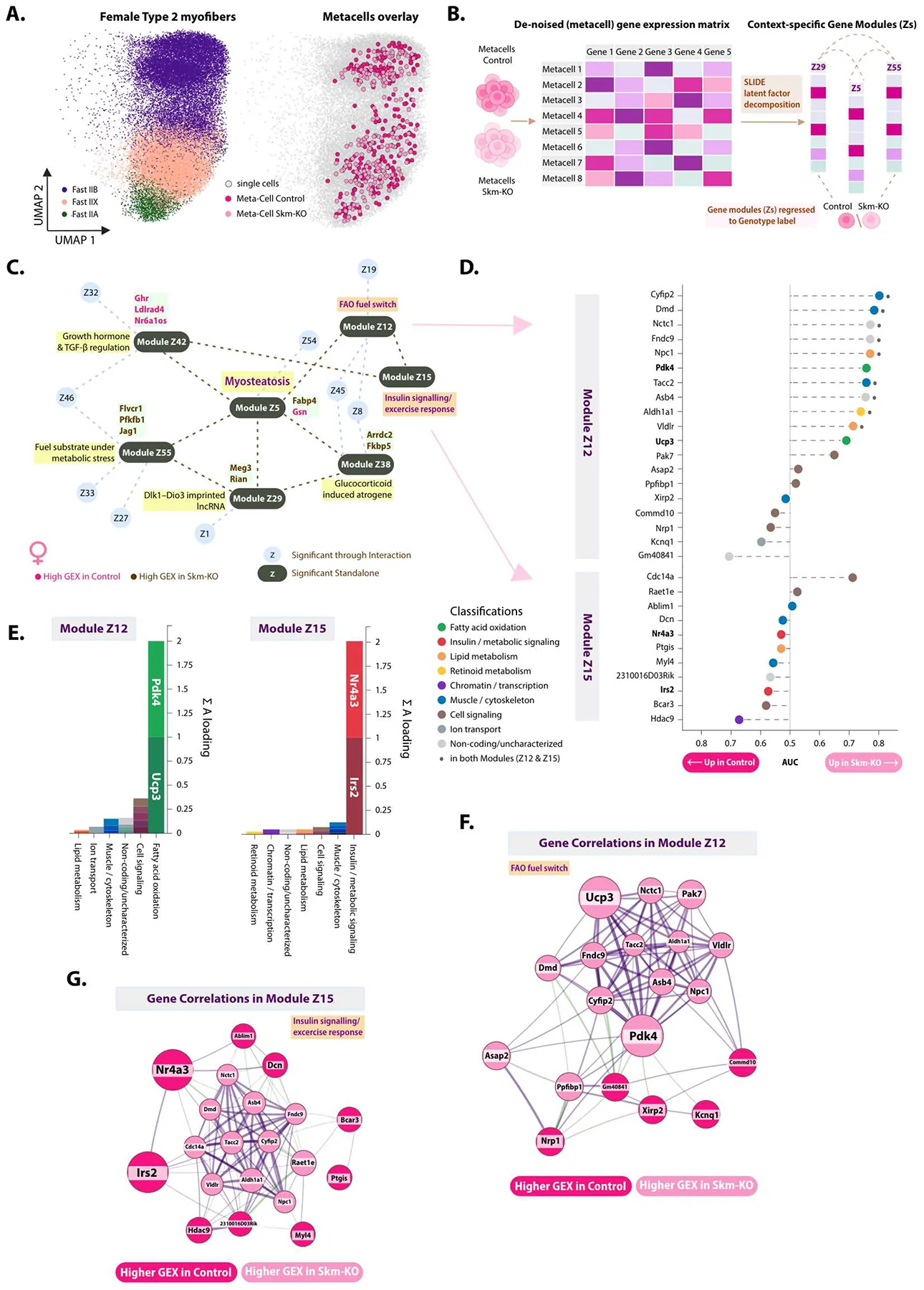
Interpretable machine learning model identifies PDK4 gene network rewiring to be associated with DNA-damage transcriptomic phenotype (A) UMAP of female Type II myofibers (Fast IIB, IIX, IIA) with denoised metacells overlaid (control and SkM-KO), used to build an imputed metacell expression matrix. (B) Schematic of the SLIDE interpretable machine-learning pipeline, where the input gene-expression matrix (450 metacells × 1,296 genes) is decomposed into context-specific latent gene modules (Z factors) that are regressed against the genotype label (control vs SkM-KO, 25-fold cross-validation, 2,000 replicates). (C) Learnt graph of the significant latent factors (standalone and interaction terms), node colour denotes higher gene expression in control (pink) versus SkM-KO (dark) and annotated by inferred function of the top 2-3 genes (highest assigned weights of the machine learning model) in the module. (D) Per-gene AUC (univariate discriminative power between the two conditions) for membership in modules Z12 (Fatty Acid Oxidation) and Z15 (Insulin Signalling/Exercise Response), ordered with AUC relative to 0.5, indicating discriminative capability of the gene in either direction. (E) Summed A-loading (model weights) per functional class for module Z12 (dominated by Pdk4/Ucp3) and module Z15 (dominated by Nr4a3/Irs2). (F, G) Gene-correlation networks of modules Z12 with PDK4 as the central hub, and Z15, node colour denotes higher expression in control versus SkM-KO, node size is scaled by A_loading. Node colours indicate functional classification of each gene as shown in the accompanying legend.

**Figure 5.**
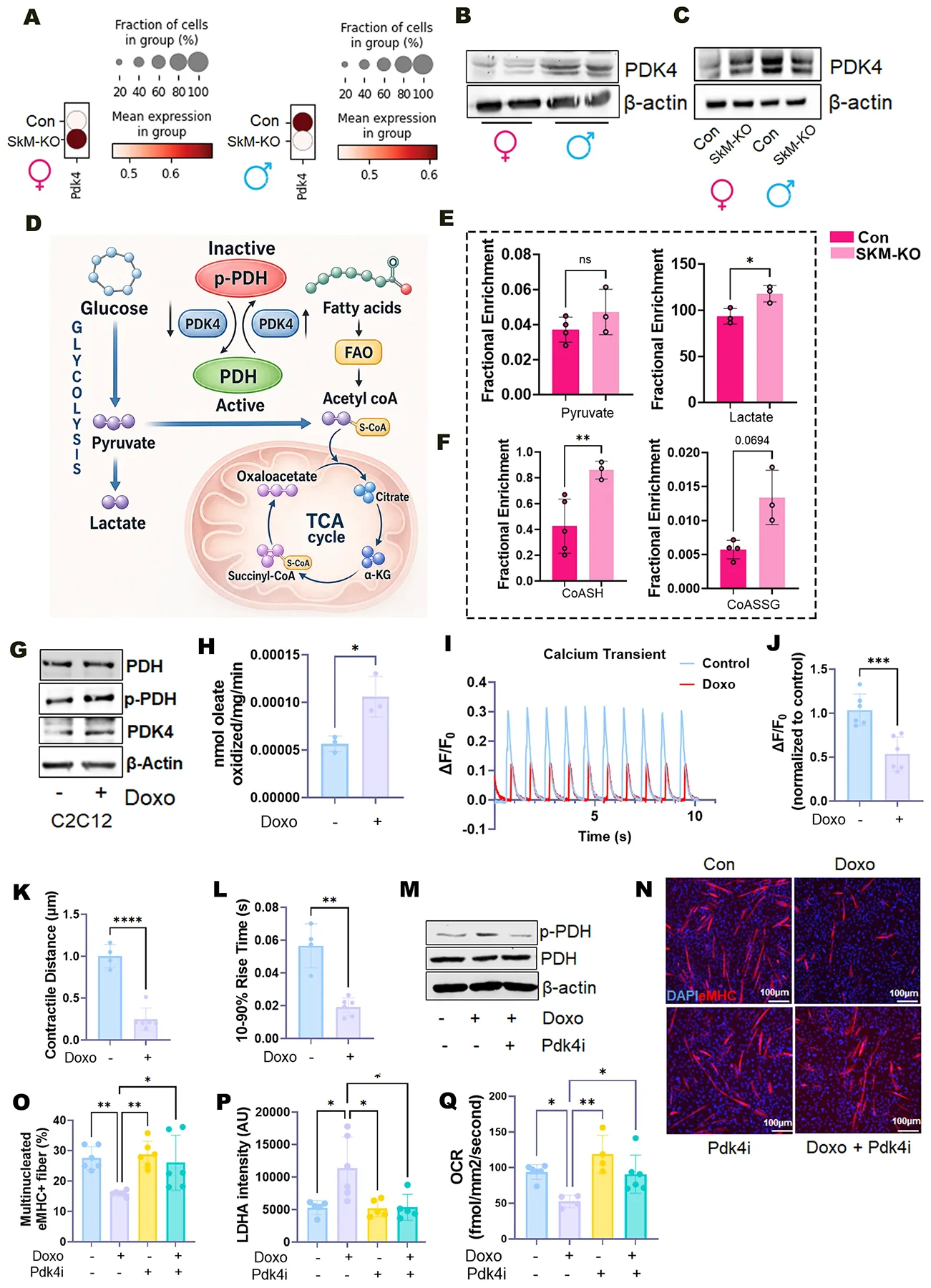
PDK4 activation promotes sex-dependent metabolic reprogramming and impaired myogenesis following DNA damage (A) snRNAseq evaluation of PDK4 in type II myonuclei. Dot size represents the fraction of meta-cells expressing Pdk4 and colour intensity indicates mean expression within each group. (B-C) Immunoblot analysis of PDK4 protein expression in control and SkM-KO TA lysates. (D) Schematic representation of PDK4-mediated regulation of glucose metabolism. PDK4 inhibits pyruvate dehydrogenase (PDH) activity by promoting PDH phosphorylation, thereby limiting pyruvate conversion to acetyl-CoA and altering substrate utilization toward fatty acid oxidation and TCA cycle metabolism. (E-F) Fractional enrichment of pyruvate, lactate, CoASH, CoASSG metabolites in control and SKM-KO female mice by LC-MS.(G) Immunoblot analysis of phosphorylated PDH (Ser 293), total PDH in C2C12 myotubes with or without doxorubicin treatment . (H) Oleate oxidation rate (nmol oleate oxidized/mg/min) in C2C12 myotubes with or without doxorubicin treatment, indicating increased fatty acid oxidation upon DNA damage. (I) Representative calcium transient traces from control and doxorubicin-treated C2C12-based three-dimensional bioengineered skeletal muscle constructs, expressed as ΔF/F_0_. (J) Quantification of calcium transient amplitude expressed as normalized ΔF/F_0_ relative to control in C2C12-based three-dimensional bioengineered skeletal muscle constructs. (K) Contractile distance (μm), representing the extent of myotube shortening during contraction in C2C12-based three-dimensional bioengineered skeletal muscle constructs. (L) Time required to reach 10–90% of the calcium transient rise (s), reflecting the kinetics of calcium activation in C2C12-based three-dimensional bioengineered skeletal muscle constructs. (M) Immunoblot analysis phosphorylated PDH and PDH following doxorubicin and/or PDK4 inhibitor treatment. (N) Representative immunofluorescence images of myosin heavy chain (MHC)-positive C2C12 myotubes following doxorubicin and/or PDK4 inhibitor treatment. Nuclei are counterstained with DAPI. Scale bars= 100 μm. (O) Quantification shows the percentage of MHC-positive multinucleated myotubes. (P) Quantification LDHA expression in C2C12 myotubes following doxorubicin and/or PDK4 inhibitor treatment. (Q) Measurement of oxygen consumption rate (OCR) in C2C12 myotubes following doxorubicin and/or PDK4 inhibitor treatment. *p<0.05, ***p<0.001, ****p<0.0001. Sample size reflected in bar plots. T-test for two-group comparison. One-way ANOVA for multiple group comparison.

**Figure 6.**
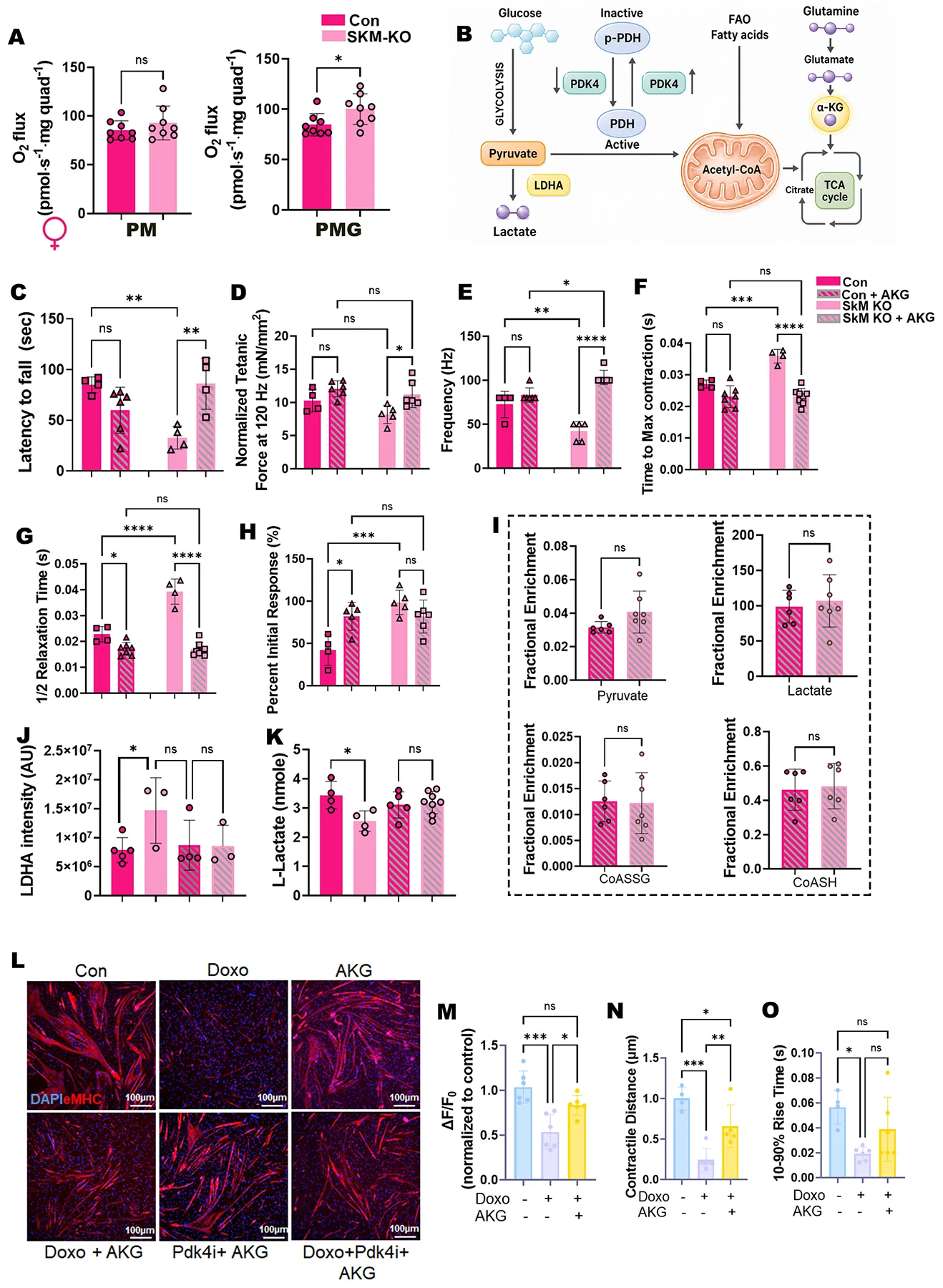
α-ketoglutarate supplementation improves skeletal muscle function and attenuates DNA damage-associated myogenic impairment. (A) High-resolution respirometry in isolated TA myofibers from female control and SkM-KO mice. Oxygen flux (pmol O_2_·s^−1^·mg quad^−1^) was measured under pyruvate + malate (PM) and pyruvate + malate + glutamate (PMG) substrate conditions, showing female SkM-KO preferential reliance on glutamate as an alternative TCA carbon source. (B) Schematic illustrating the metabolic rationale for αKG supplementation: PDK4-mediated PDC inhibition diverts pyruvate to lactate via LDHA, depleting TCA intermediates. Dietary αKG bypasses the PDC block by entering the TCA cycle as succinyl-CoA downstream of the enzymatic lesion. Mice were fed a 2% calcium αKG diet from 6 to 9 months of age. (C) Rotarod assessment. (D)Normalized Tetanic force for TA stimulation. (E) Frequency for TA stimulation. (F) Time to maximum contraction after single twitch stimulation of the TA muscle. (G) Half relaxation time of the TA muscle following single twitch stimulation. (H) Fatigue recovery expressed as percent initial response (%) following a fatigue protocol in the TA muscle. (I) Fractional enrichment of pyruvate, lactate, CoASH, CoASSG metabolites in control and SKM-KO female mice by LC-MS. (J) Quantification of LDHA expression in TA muscle sections from control and SkM-KO female mice with or without dietary AKG supplementation. (K) Serum L-lactate levels (nmole) in control and SkM-KO female mice with or without dietary αKG supplementation (L) Representative immunofluorescence images of multinucleated eMHC-positive myotubes in C2C12-based three-dimensional bioengineered muscle constructs under six treatment conditions: control, doxorubicin, αKG alone, doxorubicin + αKG, PDK4 inhibitor + αKG, and doxorubicin + PDK4 inhibitor + αKG. Nuclei counterstained with DAPI. Scale bars= 100 μm. (M–O) Quantitative analysis of calcium-handling and contractile parameters in C2C12-based engineered skeletal muscle constructs under control, doxorubicin (Doxo), and Doxo + α-ketoglutarate (αKG) conditions. (M) Calcium transient amplitude expressed as normalized ΔF/F_0_ relative to control. (N) Contractile distance (μm), representing the extent of cellular shortening during contraction. (O) Time required to reach 10–90% of the calcium transient rise (s), reflecting the kinetics of calcium activation. Treatment conditions are indicated below each graph. *p<0.05, ***p<0.001, ****p<0.0001. Sample size reflected in bar plots. *t*-test for two-group comparison. One-way ANOVA for multiple group comparison.

**Figure 7.**
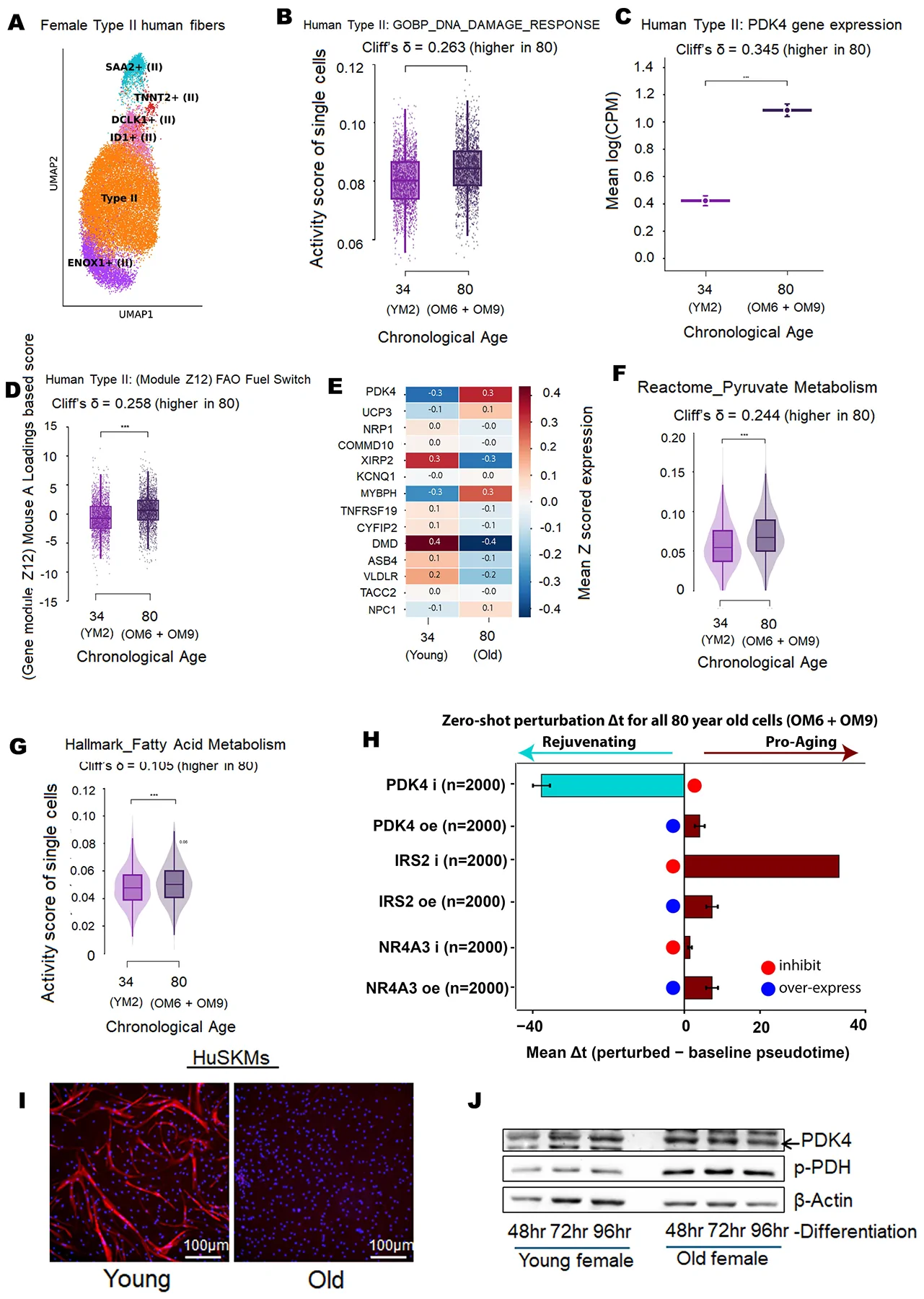
Naturally aged human skeletal muscle displays conserved signatures of metabolic reprogramming across species (A) UMAP of female human skeletal muscle type II myonuclei (3,989 nuclei retained after QC enrichment for high DNA damage burden, from an initial atlas of 13,064 type II myofiber cells). Two age groups are compared: young (age 34, YM2, n=1 donor, 2 replicates) and old (age 80, OM6+OM9, n=2 donors, 4 replicates each). Type II subpopulations are annotated by marker gene expression. (B) DNA-Damage response activity scoring of Type II myonuclei, is higher in aged (80) muscle (Cliff’s *δ* = 0.263). (C) PDK4 gene expression (mean log(CPM)) is markedly higher in aged Type II nuclei (Cliff’s *δ* = 0.345). (D-E) Cross-species transfer of mouse SLIDE Z12 module A-loadings to human type II myonuclei yields an FAO fuel-switch activity score that is significantly elevated in aged (80-year-old) versus young (34-year-old) nuclei (Cliff's δ = 0.258). Cross-species age-label prediction AUC = 0.629, indicating above-chance conservation of the genotoxic metabolic module across species and between genetic repair deficiency and natural aging. (F-G) Age-associated shifts in metabolic-pathway activity with HALLMARK REACTOME pyruvate metabolism (*δ*=0.244, higher at 80) and fatty-acid metabolism (Cliff’s *δ* = 0.105, higher at 80) increasing with natural aging. (H) Zero-shot in-silico perturbation using MaxToki, a decoder-only transformer model of cell-state aging trajectories pretrained on 175M+ human cells. For all 80-year-old type II myonuclei (OM6+OM9, n=2,000 query cells), mean Δt reflects the shift in pseudotime after perturbation relative to unperturbed baseline; negative Δt indicates movement toward a younger transcriptional state (rejuvenating) and positive Δt indicates proaging. PDK4 inhibition, IRS2 inhibition, and NR4A3 inhibition and overexpression are shown for n=2,000 query cells each. Group differences were assessed by Mann–Whitney U test with the Cliff’s *δ* effect size.***p < 0.001. (I) Representative immunofluorescence images of eMHC-positive (red) primary human skeletal muscle cells (HuSKMs) from young and old female donors during myogenic differentiation. Nuclei counterstained with DAPI. Scale bars= 100 μm. **J**. Immunoblot analysis of PDK4 and phosphorylated PDH (p-PDH Ser293) expression in primary HuSKMs from young and old female donors at 48, 72, and 96 hours of differentiation. β-Actin serves as loading control.

**Table 1:** 

Antibody/Target	Source/Catalog No.	Dilution	Complementary Secondary Antibody (Source)	Secondary Dilution
**Embryonic myosin heavy chain (eMHC)**	DSHB F1.652	1:150	Goat anti-mouse IgG1 594 (Invitrogen A21125)	1:500
**LDHA**	PA5-27406, Invitrogen	1:1000	Goat Anti-Rabbit 488	1:500
**Hoechst**	62249, Thermo Scientific	1:1000	N/A	N/A
**Flouromount-G+DAPI**	00-4959-52	N/A	N/A	N/A
**Type I**	DSHB BA-D5c	1:100	Goat anti-Mouse IgG2b 350	1:500
**Type IIA**	DSHB SC-71	1:100	Goat anti-Mouse IgG1 594	1:500
**Type IIB**	DSHB BF-F3	1:100	Goat anti-Mouse IgM 488	1:500
**Laminin**	PA1-16730, Invitrogen	1:500	Goat Anti-Rabbit 488	1:500
**PDK4**	PA5-13776, Invitrogen	1:1000	Goat Anti-Rabbit (7074s, CST)	1:10000
**PDH**	3205s, CST	1:1000	Goat Anti-Rabbit	1:10000
**p-PDH**	31866s, CST	1:1000	Goat Anti-Rabbit	1:10000
**β-Actin**	sc-47778, Santa Cruz	1:1000	Goat Anti-Mouse (7076s, CST)	1:10000

**Table 2.** 

Gene	Primer sequence (5’-3’)
Mouse p21 Forward	GTACTTCCTCTGCCCTGCTG
Mouse p21 Reverse	GTTTTCGGCCCTGAGATGTTC
Mouse Atrogin 1 Forward	TCGCAGCCAAGAAGAGAAAGAA
Mouse Atrogin 1 Reverse	GTCGAGAAGTCCAGTCTGTTGA
Mouse ERCC1 Forward	TAATAGCATCATCGTGAGCCCG
Mouse ERCC1 Reverse	TGGATGTAGTCTGGATGGAGGT
Mouse β-Actin Forward	TCGTGCGTGACATCAAAGAGA
Mouse β-Actin Reverse	GCCACAGGATTCCATACCCAA
